# Sperm‐Derived CircRNA‐1572 Regulates Embryogenesis and Zygotic Genome Activation by Targeting *CCNB2* via Bta‐miR‐2478‐L‐2

**DOI:** 10.1002/advs.202414325

**Published:** 2025-03-17

**Authors:** Yanfang Wu, Yaochang Wei, Yuelin Li, Yiming Dou, YongQiang Yang, Hanghang Liu, Xiaoyan Wang, Zheng Wang, Jianmin Su, Yong Zhang, Yongsheng Wang

**Affiliations:** ^1^ College of Veterinary Medicine Key Laboratory of Animal Biotechnology Ministry of Agriculture and Rural Affairs Northwest A&F University Yangling Shaanxi 712100 P. R. China

**Keywords:** circRNAs, cyclin B2, early embryonic development, porcine, sperm, zygotic genome activation

## Abstract

Sperm non‐coding RNAs, including micro RNAs, transfer RNA‐derived small RNAs, and long non‐coding RNAs, are pivotal in cellular cytoskeletal remodeling, early embryonic development, and offspring phenotypes. Despite the identification of circular RNAs (circRNAs) in mammals, the roles of sperm‐derived circRNAs in embryogenesis remain largely unexplored. This study identify circRNA‐1572, a sperm‐derived circRNA deliver into oocytes during fertilization, through whole‐transcriptome sequencing of porcine metaphase II (MII) oocytes, purified mature sperm, and in vitro fertilized pronuclear (PN) embryos. Functional assays confirm circRNA‐1572 competitively binds to bta‐miR‐2478‐L‐2 through a “sponge” mechanism, regulating the expression of the target gene cyclin B2 (*CCNB2*). Knockdown (KD) of circRNA‐1572 or overexpression of bta‐miR‐2478‐L‐2 led to reduce levels of CCNB2 mRNA and protein, along with altered fibrous actin (F‐actin) distribution and aberrant chromosomal organization, leading to increase developmental arrest and impair zygotic genome activation (ZGA) during early porcine embryogenesis. Importantly, these phenotypes are rescued upon supplementary mRNA of *CCNB2*. Moreover, SMART‐seq analysis reveals KD of *CCNB2* resulted in delayed degradation of maternal transcripts in 2‐cell embryos and delayed initiation of ZGA in 4‐cell. This study provides novel insights into the molecular regulatory functions of sperm‐derived circRNAs in early mammalian embryogenesis and underscores the impact of paternal factors on embryonic development.

## Graphical abstract

Sperm‐derived circRNA‐1572 competitively binds to bta‐miR‐2478‐L‐2 through a “sponge” mechanism to regulate cyclin B2 (*CCNB2*) expression, thereby modulating the distribution of fibrous actin (F‐actin) in early pig embryos, ensuring proper chromosome segregation during mitosis, and facilitating normal embryo cleavage and zygotic genome activation. This study provides novel insights into the molecular regulatory functions of sperm‐derived circular RNAs (circRNAs) in early mammalian embryogenesis.

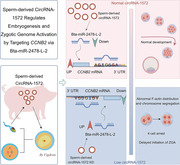

## 1 Introduction

Current research suggests that, in addition to providing the paternal haploid chromosomes and facilitating the activation of post‐fertilization embryonic development, sperm also carry epigenetic information molecules playing a significant role in regulating genomic activity and transmitting information to the next generation, thereby influencing embryonic development and offspring phenotypes.^[^ ^1^ ^]^ The significance of these epigenetic factors in offspring development has become increasingly conspicuous with the extensive exploration on the molecular mechanisms underlying the transmission of various epigenetic information between generations. Notably, non‐coding RNAs (ncRNAs) in sperm have gained significant attention, with abundant evidence indicating that certain ncRNAs, delivered by sperm into oocytes during fertilization, play a key role in preimplantation development.^[^ ^2^ ^]^ For example, bovine sperm‐derived miR‐449b is involved in embryonic epigenetic reprogramming and blastocyst apoptosis.^[^ ^3^ ^]^ Additionally, sperm‐derived miR‐202 targets SEPT7 to regulate cytoskeletal remodeling during the first cleavage of bovine embryos.^[^ ^4^ ^]^ Similarly, sperm‐borne long non‐coding RNAs (lncRNAs), Piwi‐interacting RNAs, and transfer RNA‐derived small RNAs (tsRNAs) significantly affect embryonic developmental competence and post‐birth phenotypes in offspring.^[^ ^5^, ^6^, ^7^, ^8^, ^9^ ^]^

With the in‐depth investigation of ncRNAs, circular RNAs (circRNAs), a subclass of ncRNAs, have garnered significant attention from researchers. CircRNAs are closed‐loop non‐coding RNAs formed by the back‐splicing of pre‐mRNA. The maturation of mRNA involves a series of consecutive processes, including transcription, splicing, capping, polyadenylation, export, and surveillance.^[^ ^10^ ^]^ Essentially, back‐splicing is coupled with transcription. Current research reveals that the molecular regulatory mechanisms of circRNAs primarily involve participation in transcriptional regulation within the nucleus, competition with mRNA precursors during transcription, acting as “sponges” for microRNAs (miRNAs), facilitating the translation of functional peptides or proteins, and interacting with proteins.^[^ ^11^ ^]^ Notably, in the field of mammalian reproduction, circRNAs may play crucial roles in male reproduction, follicle development, oocyte maturation, and preimplantation embryo development, as elucidated through sequencing and analysis of circRNAs in gametes,^[^ ^12^ ^]^ embryos,^[^ ^13^ ^]^ testes,^[^ ^14^ ^]^ ovaries,^[^ ^15^ ^]^ uterus,^[^ ^16^ ^]^ and placenta.^[^ ^17^ ^]^ However, these studies have primarily focused on circRNA screening and expression patterns, and an in‐depth exploration of circRNA functions remains lacking. In 2019, Shen et al. reported that circRNAs act as miRNA sponges to regulate GnRH signaling and oocyte maturation,^[^ ^18^ ^]^ Additionally, two other studies demonstrated that two different circRNAs may act as miRNA sponges, affecting blastocyst quality and embryonic developmental potential in pigs.^[^ ^19^, ^20^ ^]^ Although circRNA expression in testicular tissue,^[^ ^21^ ^]^ semen,^[^ ^22^ ^]^ and sperm^[^ ^23^ ^]^ has been identified, and Chioccarelli et al. reported that the biogenesis of sperm circCNOT6L in mice and humans supports zygote development,^[^ ^24^ ^]^ a more in‐depth investigation into the role of sperm‐derived circRNAs in mammalian spermatogenesis and embryonic development has not been reported. Therefore, research on the impact of sperm‐derived circRNAs on early embryonic development and the mechanisms by which they exert their regulatory effects is imperative.

In mammalian oocytes, maternal mRNAs are transcribed and stored during oocyte growth. These mRNAs support oocyte meiotic maturation and early embryo development but undergo widespread degradation following meiotic resumption. Subsequently, the zygotic genome is activated for transcription, marking the transition from maternal to zygotic developmental control, known as the maternal‐to‐zygotic transition (MZT).^[^ ^25^, ^26^, ^27^ ^]^ Notably, during MZT, approximately 60% of maternal mRNA levels significantly decreased in fruit flies,^[^ ^28^ ^]^ whereas in mice, up to 90% of maternal mRNA is eliminated at the 2‐cell stage.^[^ ^29^ ^]^ Zygotic genome activation (ZGA) is a pivotal phase in the pre‐implantation development of mammalian embryos, characterized by an increase in gene expression. The initiation of embryonic development relies heavily on the expression products of the zygotic genome. ZGA typically occurs during the 2‐cell stage in mice, the 4‐cell stage in pigs, and the 4‐cell to 8‐cell stages in cattle and humans.^[^ ^30^ ^]^ Research has demonstrated a robust correlation between the duration of embryonic developmental arrest and the timing of ZGA. For instance, studies by Sun indicate that the arrest of 2‐cell mouse embryos coincides with suppressed transcriptional activity,^[^ ^31^ ^]^ and Zhang's investigations reveal that under nutrient‐deficient conditions, most pig embryos arrest at the 4‐cell stage, resulting in failed ZGA.^[^ ^32^ ^]^ Additionally, aberrant clearance of maternal mRNA is detrimental to ZGA, leading to embryonic developmental stasis and pregnancy failure.^[^ ^33^ ^]^ Consequently, the harmonious coordination between the degradation of maternal substances and ZGA during the MZT is crucial for normal embryonic development.

Early embryonic cleavage is a continuous and rapid process akin to mitosis. During the initial stages of embryonic development, cell cycle regulatory factors stored in oocytes are swiftly activated after fertilization, bypassing the G1 and G2 phases and transitioning directly from the S phase (DNA replication) to the M phase (mitosis).^[^ ^34^ ^]^ Among these regulatory factors are cyclin B2 (CCNB2) and cyclin B1 (CCNB1), members of the cyclin B family. The cyclin‐dependent kinase 1 (Cdk1)–cyclin B regulatory network plays a well‐recognized role in mitotic entry.^[^ ^35^ ^]^ Additionally, Cdk1–cyclin B is involved in nuclear envelope breakdown, chromosome condensation, spindle assembly, regulation of the spindle assembly checkpoint, and inhibition of separase activity.^[^ ^36^, ^37^, ^38^, ^39^, ^40^ ^]^ Historically, the CCNB1/CDK1 complex was considered the primary driver of cell division processes, with CCNB1 being more crucial for cell proliferation (at least in mitosis) than cyclin B2. However, recent research has highlighted an equally important role for cyclin B2 in cell division.^[^ ^41^, ^42^, ^43^, ^44^ ^]^ Despite extensive studies on the function of cyclin B2 in mitosis and oocyte meiosis, its role in early embryonic development remains unknown.

In this study, for the first time, we delved into the role and mechanism of sperm‐derived circRNAs in regulating early embryonic development in pigs. We identified circRNA‐1572, which is transferred from sperm into the oocyte during fertilization. A reduction in the expression of circRNA‐1572 led to developmental arrest and the inhibition of ZGA during porcine embryogenesis. Furthermore, we elucidated that sperm‐derived circRNA‐1572 competitively binds to bta‐miR‐2478‐L‐2 through a “sponge” mechanism to target and regulate cyclin B2 expression, thereby modulating the distribution of fibrous actin (F‐actin) in early pig embryos, ensuring proper chromosome segregation during mitosis, and facilitating normal embryo cleavage, maternal RNA degradation (MRD), and ZGA. Our findings offer novel perspectives into the influence of sperm‐derived RNA on embryonic development and its molecular regulatory mechanisms, thereby enriching the research framework on the regulatory role of sperm‐derived circRNAs in early embryonic development.

## 2 Results

### 2.1 Screening of Sperm‐Derived RNA

**Figure 1:**
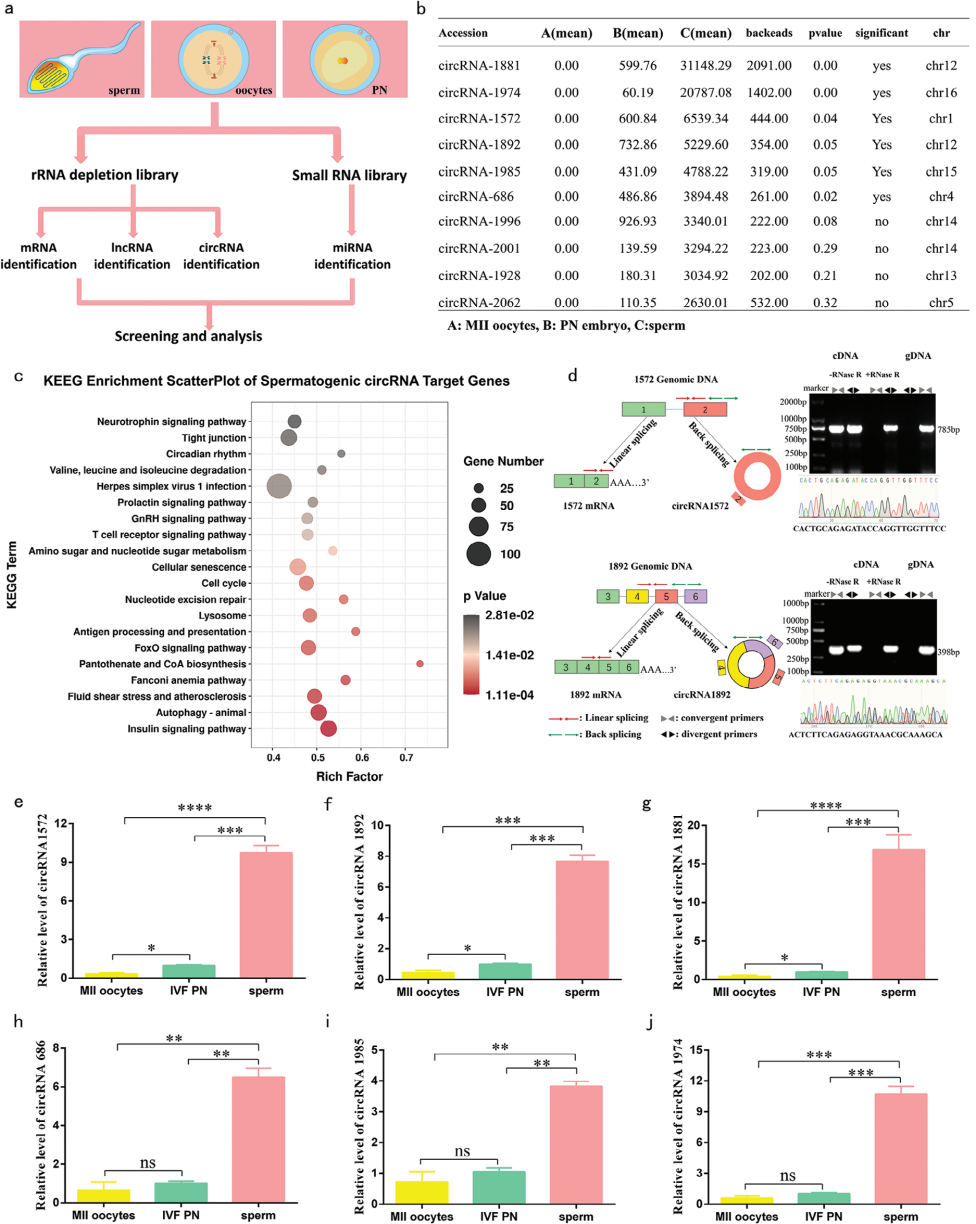
Identification of sperm‐derived circRNAs. a) The primary workflow of whole transcriptome sequencing analysis of MII oocytes, PN embryos, and sperm. b) Sperm‐derived circRNA information. c) KEEG analyses of Sperm‐derived circRNA target genes (A, MII oocytes; B, PN embryos; C, sperm). d) Genomic regions of the circRNA genes derived from various exons of circRNA‐1572, circRNA‐1892 (left). Convergent (gray) and divergent (black) primers used for amplification of linear or back‐spliced products, RT‐PCR of total RNA from RNase R‐treated or untreated sperm (top right), and further validation through Sanger sequencing (bottom right), all these demonstrated circular structure of the circRNAs. e–j) Relative expression of circRNA‐1572, circRNA‐1892, circRNA‐1881, circRNA‐686, circRNA‐1985, and circRNA‐1974 in oocytes, sperm, and IVF PN embryos, with total RNA extracted and detected using RT‐qPCR. The data are presented as mean ± SEM from at least three independent experiments., * *p* < 0.05, ** *p*< 0.01, *** *p* <0.001, **** *p* <0.0001, ns: not significant.

Following the collection of porcine metaphase II (MII) oocytes, purified mature sperm, pronuclear (PN) 1‐cell embryos, total RNA were extracted and subjected to rRNA depletion to construct strand‐specific small RNA libraries. Based on these libraries, mRNA, lncRNA, circRNA, and miRNA were analyzed (**Figure** **1**a). Through rigorous screening, we obtained 81 321 510 (94.11% of the total clean data), 79 663 020 (92.58% of the total clean data), and 79 419 631 (93.34% of the total clean data) high‐quality reads from porcine MII oocytes, PN embryos, and sperm, respectively, and the proportion of bases with a quality value ≥30 was 96.84%, 97.30%, and 97.12%, respectively (Table S1, Supporting Information), indicating the accuracy and efficacy of our sequencing data.

We performed differential gene expression analysis for mRNA, lncRNA, circRNA, and miRNA to identify the sperm‐derived RNAs that enter the oocyte. The results were filtered and organized based on the principle of high expression in sperm and PN embryos, but not in MII oocytes. Notably, during the screening of mRNA and lncRNA, when the expression level in MII oocytes was set to 0, the expression level in PN embryos was also nearly 0, while expression was evident in sperm, the mRNA and lncRNA expression patterns in MII oocytes and PN embryos were similar but distinct from those in sperm (Figure S1a–d, Supporting Information), suggesting a lack of apparent delivery of mRNAs or lncRNAs from sperm to oocytes during porcine fertilization.

We successfully identified several sperm‐derived miRNAs (Figure S1e,f, Supporting Information) and circRNAs (Figure 1b). Previous studies have verified the presence of sperm‐derived miRNAs and their contributions to embryonic development,^[^ ^3^, ^4^ ^]^ but the impact of sperm‐derived circRNAs on early mammalian embryonic development remains unknown. Therefore, our research focus was redirected toward sperm‐derived circRNAs. Gene Ontology (GO) enrichment analysis of target genes of sperm‐derived circRNAs (Figure S1g. Supporting Information) revealed their participation in cell adhesion molecules, RNA polymerase II intronic transcription regulatory region sequence‐specific DNA binding, mismatch repair, and chromosome centromeric regions. Furthermore, Kyoto Encyclopedia of Genes and Genomes (KEGG) enrichment analysis (Figure 1c) indicated their involvement in pantothenate and CoA biosynthesis, the cell cycle, and nucleotide excision repair. Taking into account their expression levels in sperm and PN embryos, along with the characteristics of back – splicing junctions and significant differential expression, we shortlisted six circRNAs for in – depth investigation (Figure 1b, top six rows): circRNA – 1572, circRNA – 1892, circRNA – 1881, circRNA – 686, circRNA – 1985, and circRNA – 1974.

### 2.2 Validation of the Circular Structure and Sperm‐Derived Origin of the Six CircRNAs

We first conducted bioinformatic annotation of the six circRNAs (Table S2, Supporting Information) and illustrated their structural patterns. To confirm the circular structures, two pairs of primers—divergent and convergent—were designed for each circRNA. PCR coupled with agarose gel electrophoresis showed that divergent primers could amplify the circular isoforms of circRNAs from cDNA but not from genomic DNA (gDNA). In contrast, convergent primers generated the corresponding linear fragments from both cDNA and gDNA. Subsequent Sanger sequencing of the PCR products further validated the sequence accuracy. Additionally, porcine sperm was subjected to RNase R treatment to assess the stability of the circRNAs, confirming their authenticity and circular configuration (circRNA‐1572 and circRNA‐1892 showed in Figure 1d, circRNA‐1974, circRNA‐1881, circRNA‐686 and circRNA‐1985 showed in Figure S1h, Supporting Information).

Quantification of the relative expression levels of the six circRNAs were then detected using qPCR. Remarkably, circRNA‐1572 (Figure 1e), circRNA‐1892 (Figure 1f), and circRNA‐1881 (Figure 1g) demonstrated minimal presence in MII oocytes, exhibiting significantly lower expression compared to PN embryos and sperm (with sperm displaying notably higher expression levels). Although circRNA‐686 (Figure 1h), circRNA‐1985 (Figure 1i), and circRNA‐1974 (Figure 1j) exhibited minimal expression in MII oocytes and high expression in sperm, the differences in their expression between MII oocytes and PN embryos were not statistically significant. We hypothesize that the discrepancy may be attributed to the sufficient RNA yield obtained for whole transcriptome sequencing across various samples, whereas the RNAs collected for qPCR analysis were limited (15–20 oocytes or embryos). Therefore, taking a comprehensive approach, we further selected circRNA‐1572, circRNA‐1892, and circRNA‐1881, which exhibited high expression in sperm, marginal presence in oocytes, and significantly higher levels in embryos compared to oocytes. Notably, designing fluorescence in situ hybridization (FISH) probes and specific siRNAs were challenging for circRNA‐1881 owing to a relatively low GC content at its circularization site. Consequently, conducting molecular functional studies on this circRNA was difficult, leading to the prioritization of circRNA‐1572 and circRNA‐1892 for further investigation.

### 2.3 Knocking Down Sperm‐Derived CircRNA‐1572 Arrests Embryogenesis and Inhibits ZGA

**Figure 2:**
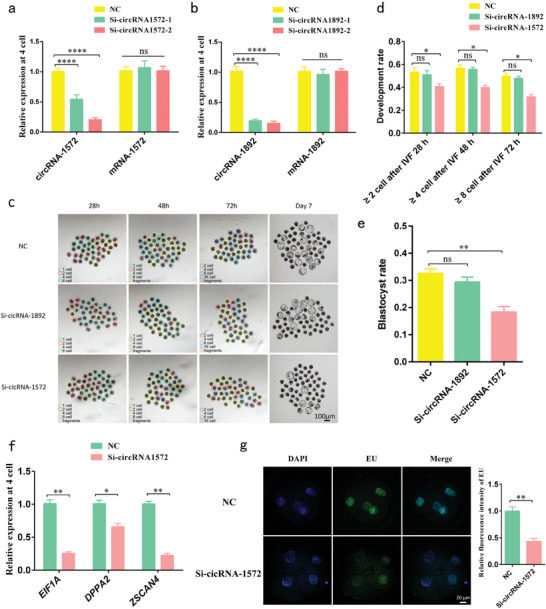
Impact of downregulating sperm‐derived circRNA‐1572 and circRNA‐1892 expression during early development in porcine IVF embryos. a, b) Validation of the efficiency of circRNA‐1572 and circRNA‐1892 KD using qRT‐PCR in samples collected from 4‐cell embryos. c, d) Comparison of embryonic development at different stages between groups (The fertilized oocytes were not deliberately selected for the presence of a second polar body, NC group: *n* = 426; Si‐circRNA‐189 group: *n* = 378; Si‐circRNA‐1572 group: *n* = 363; scale bar = 100 µm). e) Blastocyst rate in different groups (NC group: *n* = 285; Si‐circRNA‐189 group: *n* = 248; Si‐circRNA‐1572 group: *n* = 200). f) Relative expression of genes (*EIF1A, DPPA2*, and *ZSCAN4*) associated with porcine zygotic genome activation at 4‐cell stage upon downregulation of circRNA‐1572 expression. g) Incorporation of EU at the 4‐cell stage upon downregulation of circRNA‐1572 expression (*n* ≥ 10 for each group, scale bar = 20 µm). The data are presented as mean ± SEM from at least three independent experiments. *p < 0.05, **p < 0.01, ****p < 0.0001, ns: not significant.

Two siRNAs targeting circRNA‐1572 or circRNA‐1892 were individually microinjected into freshly fertilized eggs following IVF (co‐incubation of sperm and eggs for 6 h). Both Si‐circRNA‐1572‐1 and Si‐circRNA‐1572‐2 significantly downregulated circRNA‐1572 expression without affecting that of its mRNA. As Si‐circRNA‐1572‐2 exhibited more pronounced interference (**Figure** **2**a), subsequent circRNA‐1572 KD experiments were conducted using Si‐circRNA‐1572‐2 alone. As for circRNA‐1892, both siRNAs demonstrated nearly equal and effective suppression of circRNA‐1892 expression without affecting that of its linear mRNA (Figure 2b) and therefore, a mixture of Si‐circRNA‐1892‐1 and Si‐circRNA‐1892‐2 was employed for subsequent circRNA‐1892 KD experiments.

At 28 h post the start of IVF, the proportion of embryos at the ≥2‐cell stage in both the negative control (NC) group and Si‐circRNA‐1892 group was comparable, at 53.44% and 51.01%, respectively, both significantly higher than the Si‐circRNA‐1572 group at 40.74% (numbers of oocytes with second polar bodies following IVF as the denominators). At 48 h post IVF initiation, the proportion of embryos at the ≥4‐cell stage in the NC group and Si‐circRNA‐1892 group was similar at 56.63% and 55.73%, respectively, again significantly higher than the Si‐circRNA‐1572 group at 39.95%. At 72 h post IVF initiation, the proportion of embryos at the ≥8‐cell stage in the NC group and Si‐circRNA‐1892 group was comparable, at 49.91% and 47.88%, respectively, both significantly higher than the Si‐circRNA‐1572 group at 31.79%, where ≈50% of the embryos were still arrested at the 4‐cell stage (Figure 2c,d; Table S3, Supporting Information). On day 7 post IVF, the blastocyst rate in the NC group was slightly higher than that in the Si‐circRNA‐1892 group, at 32.61% and 29.44%, respectively, both significantly higher than the Si‐circRNA‐1572 group at 18.39% (Figure 2e). These results indicate that KD of circRNA‐1572, rather than circRNA‐1892, predominantly arrests embryos at the 4‐cell stage and affects blastocyst formation.

Coincidentally, ZGA in pigs also begins at the 4‐cell stage. To further investigate whether circRNA‐1572 KD simultaneously affects ZGA, we analyzed key marker genes related to pig ZGA. Compared with the NC group, KD of circRNA‐1572 via Si‐circRNA‐1572 significantly decreased *EIF1A*, *DPPA2*, and *ZSCAN4* mRNA levels in 4‐cell embryos (Figure 2f). Additionally, the 5‐ethynyl uridine (EU) RNA incorporation assay revealed a significant decrease in EU incorporation in KD embryos, indicating a substantial reduction in transcription during ZGA (Figure 2g). These findings suggest that KD of circRNA‐1572 arrests pig IVF embryo development and inhibits ZGA.

### 2.4 Sperm‐Derived CircRNA‐1572 Exerts Targeted Regulation on Bta‐miR‐2478‐L‐2 Through Sponge Adsorption

**Figure 3:**
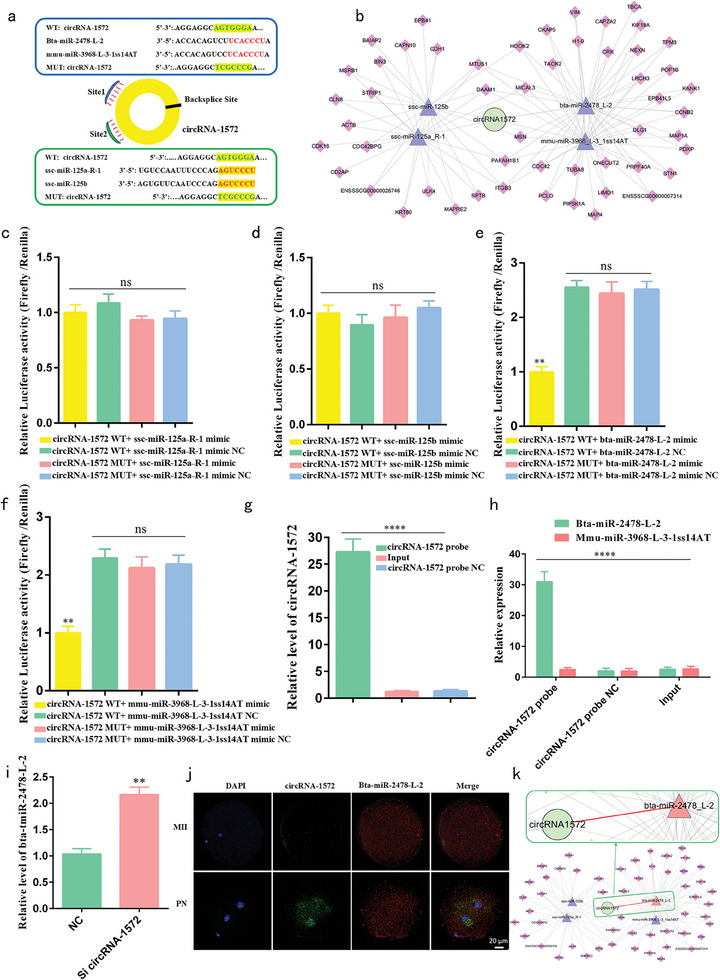
Sperm‐derived circRNA‐1572 mediates targeted regulation of miR‐2478‐L‐2 through sponge adsorption. a) Predicted binding sites of four miRNAs (bta‐miR‐2478‐L‐2, mmu‐miR‐3968‐L‐3‐1ss14AT, ssc‐miR‐125a‐R‐1, and ssc‐miR‐125b) on circRNA‐1572. b) Network diagram of the circRNA‐1572 – miRNA – mRNA interactions. c–f) Luciferase activity analysis upon co‐transfection of mimic or mimic NC of the four miRNAs with the circRNA‐1572‐WT or circRNA‐1572‐MUT plasmids. g, h) RNA pulldown assay showing significant enrichment of circRNA‐1572 and bta‐miR‐2478‐L‐2 in the biotin‐labeled circRNA‐specific probe group. i) Relative expression of bta‐miR‐2478‐L‐2 after downregulation of circRNA‐1572 expression. j) In situ hybridization of circRNA‐1572 and bta‐miR‐2478‐L‐2 in porcine MII oocytes and PN embryos (2 pronuclei) (*n* ≥ 10 for each group, scale bar = 20 µm). h) Network diagram of the circRNA‐1572 – bta‐miR‐2478‐L‐2. The data are presented as mean ± SEM from at least three independent experiments. **p < 0.01, ****p < 0.0001, ns: not significant.

Computational predictions using TargetScan and MiRanda software identified 14 miRNAs as potential targets of circRNA‐1572. Among these, bta‐miR‐2478‐L‐2, mmu‐miR‐3968‐L‐3‐1ss14AT, ssc‐miR‐125a‐R‐1, and ssc‐miR‐125b ranked among the top four in relative expression levels in PN embryos as determined by miRNA library analysis. Both bta‐miR‐2478‐L‐2 and mmu‐miR‐3968‐L‐3‐1ss14AT were predicted to share the same binding site (site 1) with circRNA‐1572, while ssc‐miR‐125a‐R‐1 and ssc‐miR‐125b were predicted to share another binding site (site 2) (**Figure** **3**a). Further, a regulatory network diagram of circRNA‐1572 was constructed based on these findings (Figure 3b).

To confirm the interactions between circRNA‐1572 and its target miRNAs, luciferase reporter assays were performed. Fragments containing binding sites 1 and 2 from circRNA‐1572, as well as the corresponding mutated sites, were inserted downstream of the luciferase coding region. Co‐transfection of mimic or mimic NC of the four miRNAs with circRNA‐1572‐wild‐type (WT) or circRNA‐1572‐mutant (MUT) into porcine fibroblasts showed that, compared with mimic NC, co‐transfection of circRNA‐1572‐WT with bta‐miR‐2478‐L‐2 mimic and mmu‐miR‐3968‐L‐3‐1ss14AT mimic significantly reduced luciferase reporter gene activity (Figure 3c–f).

Additionally, RNA pulldown assays were performed using biotin‐labeled circRNA‐specific probes. Notably, specific enrichment of circRNA‐1572 (Figure 3g) and bta‐miR‐2478‐L‐2 was observed in the circRNA‐1572 probe group (upregulated 27.33 and 32.6 times, respectively), whereas miR‐3968‐L‐3‐1ss14AT was not detected (Figure 3h). Additionally, KD of circRNA‐1572 significantly increased the relative expression of bta‐miR‐2478‐L‐2 at the 4‐cell stage (Figure 3i). Subsequently, based on the circRNA‐1572 sequence and structure, we designed RNA FISH probes and performed FISH localization detection. Notably, circRNA‐1572, transmitted into the oocyte by sperm, exhibited cytoplasmic localization in porcine IVF PN embryos, while it was not detected in MII oocytes. Furthermore, there are some co‐localizations of circRNA‐1572 and bta‐miR‐2478‐L‐2 in the cytoplasm of PN embryos (Figure 3j). Therefore, all these results collectively indicate that circRNA‐1572 targets bta‐miR‐2478‐L‐2(Figure 3k).

### 2.5 Sperm‐Derived CircRNA‐1572 Targets and Regulates CCNB2 via Bta‐miR‐2478‐L‐2

**Figure 4:**
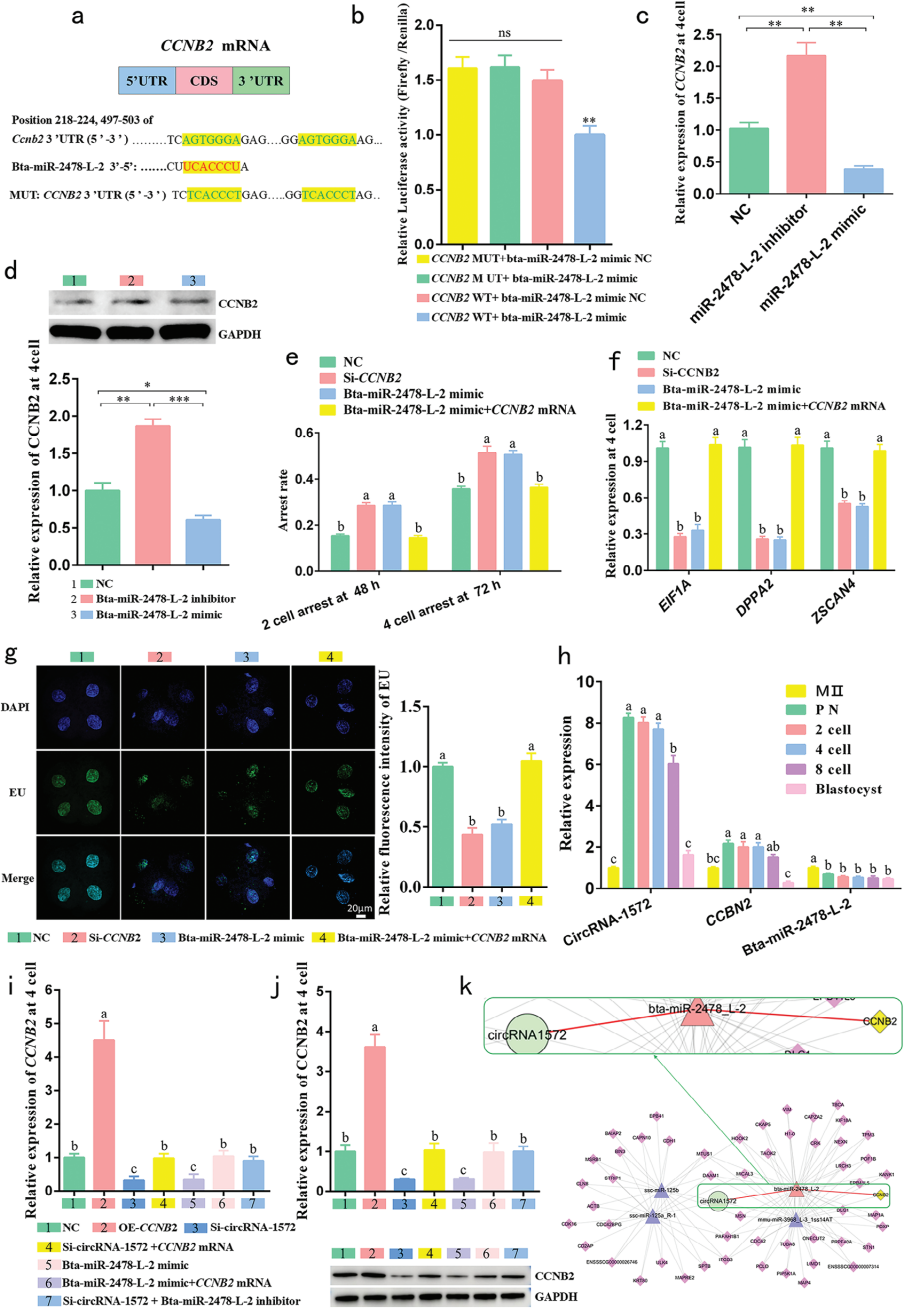
Sperm‐derived circRNA‐1572 regulates *CCNB2* by targeting bta‐miR‐2478‐L‐2. a) Schematic representation of the binding sites between *CCNB2* 3ʹ UTR and bta‐miR‐2478‐L‐2. b) Luciferase activity analysis upon co‐transfection of bta‐miR‐2478‐L‐2 mimic or mimic NC with the *CCNB2*‐WT or *CCNB2*‐MUT plasmids. c, d) Analysis of *CCNB2* mRNA (each group comprised 30 embryos) and CCNB2 protein (each group comprised 200 embryos) expression levels at 4‐cell after injection of bta‐miR‐2478‐L‐2 mimic (overexpression group) and bta‐miR‐2478‐L‐2 inhibitor (inhibition group). e) Embryonic arrest at the 2‐ and 4‐cell stages after injection of bta‐miR‐2478‐L‐2 mimic, Si‐*CCNB2* (KD group). f) Relative expression of genes (*EIF1A, DPPA2*, and *ZSCAN4*) associated with porcine zygotic genome activation upon injection of bta‐miR‐2478‐L‐2 mimic, Si‐*CCNB2* and co‐injection of bta‐miR‐2478‐L2 mimic and *CCNB2* mRNA. g) Incorporation of EU at the 4‐cell stage after injection of Si‐*CCNB2*, bta‐miR‐2478‐L‐2 mimic and co‐injection of bta‐miR‐2478‐L2 mimic + *CCNB2* mRNA (*n* ≥6 for each group, scale bar = 20 µm). h) Relative expression of circRNA‐1572, bta‐miR‐2478‐L‐2, and *CCNB2* at different stages of IVF early embryos (1‐, 2‐, 4‐, 8‐cell, and blastocyst). i, j) Analysis of *CCNB2* mRNA and protein expression levels at 4‐cell after injection of *CCNB2* mRNA, Si‐circRNA‐1572, bta‐miR‐2478‐L‐2 mimic, co‐injection of Si‐circRNA‐1572 + CCNB2 mRNA, co‐injection of bta‐miR‐2478‐L‐2 mimic + *CCNB2* mRNA and co‐injection of Si‐circRNA‐1572 + bta‐miR‐2478‐L‐2 inhibitor. (K) Network diagram of the circRNA‐1572 – bta‐miR‐2478‐L‐2 –*CCNB2*. The data are presented as mean ± SEM from at least three independent experiments. *p < 0.05, **p < 0.01, different lowercase letters (a, b, and c) indicate significant difference between groups, *p* < 0.05, ns: not significant.

Target genes of bta‐miR‐2478‐L‐2 were predicted using TargetScan and MiRanda software. Considering the embryonic developmental arrest, we hypothesized that CCNB2 may be involved. The 3ʹ untranslated region (3ʹ UTR) of *CCNB2* harbors two identical binding sites for bta‐miR‐2478‐L‐2 (**Figure** **4**a). Subsequently, we constructed WT and MUT luciferase reporter plasmids containing these binding sites and performed dual‐luciferase assays. Notably, co‐transfection of WT and bta‐miR‐2478‐L‐2 mimics significantly reduced luciferase activity (Figure 4b). Moreover, qPCR and western blotting results demonstrated that microinjection of bta‐miR‐2478‐L‐2 mimic or inhibitor significantly downregulated or upregulated, respectively, the mRNA (Figure 4c) and protein levels of CCNB2 at the 4‐cell stage (Figure 4d), affirming *CCNB2* as a direct target of bta‐miR‐2478‐L‐2. Furthermore, *CCNB2* KD via Si‐*CCNB2* or bta‐miR‐2478‐L‐2 overexpression via the mimic resulted in a significant increase in the proportion of embryos arrested at the 2‐cell and 4‐cell stages (Figure 4e), along with notable downregulation of ZGA marker genes *EIF1A*, *DPPA2*, and *ZSCAN4* mRNA levels (Figure 4f) and a notable reduction in the newly generated RNA at the 4‐cell stage (Figure 4g). Furthermore, co‐injection of bta‐miR‐2478‐L2 mimic and *CCNB2* mRNA alleviated this condition, approaching the NC group's trend. In conclusion, bta‐miR‐2478‐L‐2 negatively modulates early cleavage and ZGA in porcine IVF embryos by targeting *CCNB2*.

To further investigate the interplay between circRNA‐1572, bta‐miR‐2478‐L‐2, and *CCNB2*, we assessed their relative expression levels at different stages from MII to blastocyst embryos using qPCR. Notably, the endogenous levels of *CCNB2* significantly increased with circRNA‐1572 from the MII stage to the PN stage, maintained high levels until the 8‐cell stage, and then significantly decreased in the blastocyst stage, whereas the trend of bta‐miR‐2478‐L‐2 was inversely correlated (Figure 4h). Subsequently, in the Western blot analyses, compared to the NC, both mRNA and protein at the 4 cell stage levels of CCBN2 significantly decreased in the injection of Si‐circRNA‐1572 and bta‐miR‐2478‐L‐2 mimic groups, while a marked increase was observed in the injection *CCNB2* mRNA group, whereas in the Si‐circRNA‐1572 + *CCNB2* mRNA, bta‐miR‐2478‐L‐2 mimic + *CCNB2* mRNA, and si‐circRNA‐1572 + bta‐miR‐2478‐L‐2 inhibitor groups, the mRNA (Figure 4i) and protein (Figure 4j) levels of CCBN2 rose to a level comparable to that of the NC group. Therefore, circRNA‐1572 regulates *CCNB2* through bta‐miR‐2478‐L‐2 during early porcine embryonic development (Figure 4k).

### 2.6 The Suppression of Bta‐miR‐2478‐L‐2 by CircRNA‐1572 is Essential for Preimplantation Development in Porcine IVF Embryos

**Figure 5:**
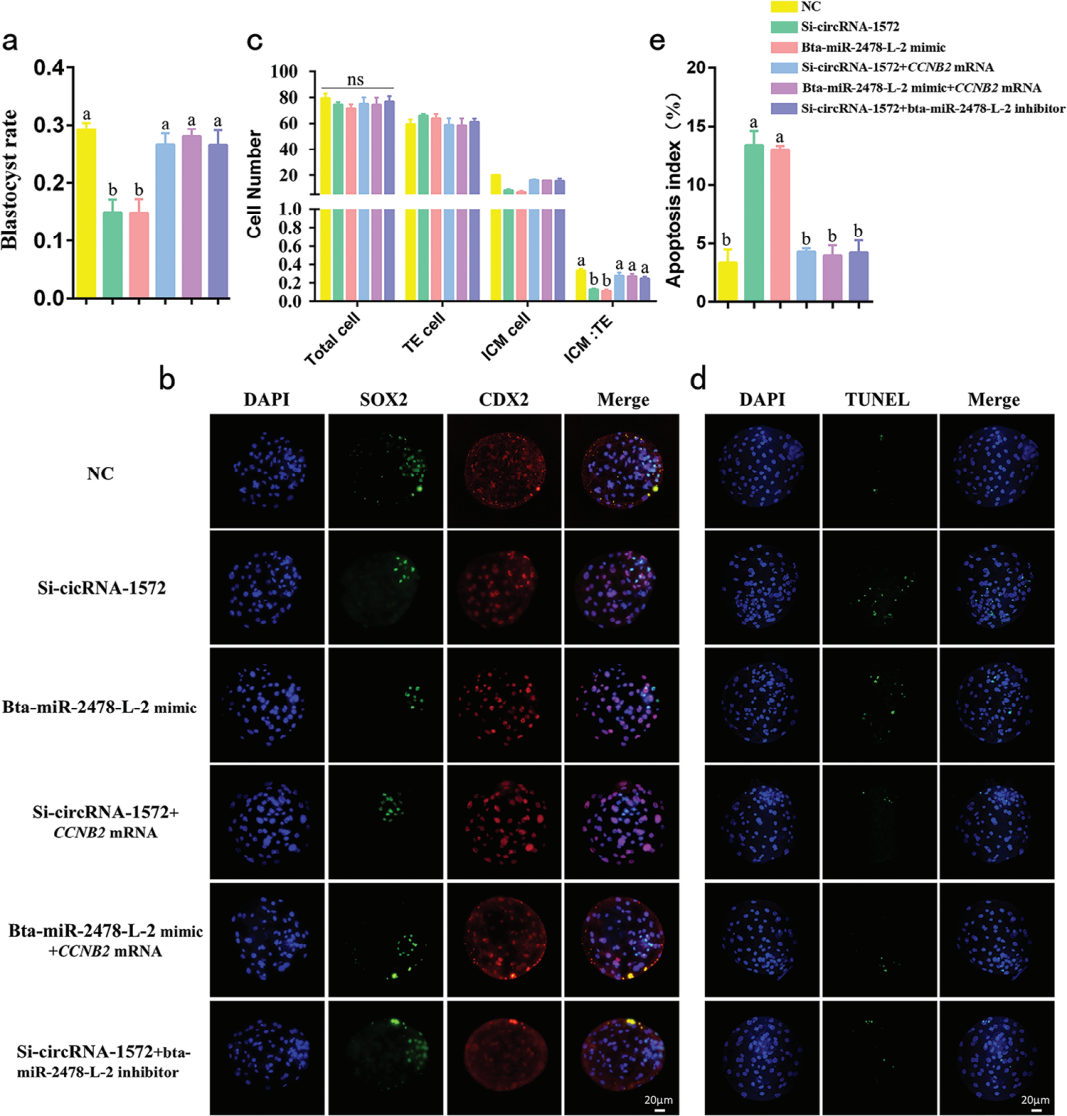
Sperm‐derived circRNA‐1572 regulates the early embryonic developmental potential by targeting *CCNB2* via bta‐miR‐2478‐L‐2. a) Blastocyst formation rates of IVF embryos after injection of Si‐circRNA‐1572, bta‐miR‐2478‐L‐2 mimic, Si‐circRNA‐1572 + CCNB2 mRNA, bta‐miR‐2478‐L‐2+ CCNB2 mRNA, Si‐circRNA‐1572 + bta‐miR‐2478‐L‐2 inhibitor, and NC (NC group: *n* = 163, Si‐circRNA‐1572 group: *n* = 171, bta‐miR‐2478‐L‐2 mimic group: *n* = 159, Si‐circRNA‐1572 + CCNB2 mRNA group: *n* = 155, bta‐miR‐2478‐L‐2+ CCNB2 mRNA group: *n* = 167, Si‐circRNA‐1572 + bta‐miR‐2478‐L‐2 inhibitor group: *n* = 146). b, c) Cell lineage staining and IVF blastocyst analysis after injection of Si‐circRNA‐1572, bta‐miR‐2478‐L‐2 mimic, Si‐circRNA‐1572 + CCNB2 mRNA, bta‐miR‐2478‐L‐2+ CCNB2 mRNA, Si‐circRNA‐1572 + bta‐miR‐2478‐L‐2 inhibitor, and NC (*n* ≥ 6 for each group, scale bar = 20 µm). d, e) Apoptosis staining and analysis of IVF blastocysts after injection of Si‐circRNA‐1572, bta‐miR‐2478‐L‐2 mimic, Si‐circRNA‐1572 + CCNB2 mRNA, bta‐miR‐2478‐L‐2 mimic+ CCNB2 mRNA, Si‐circRNA‐1572 + bta‐miR‐2478‐L‐2 inhibitor, and NC (*n* ≥ 10 for each group, scale bar = 20 µm). The data are presented as mean ± SEM from at least three independent experiments. Different lowercase letters a, b) indicate significant difference between groups, *p* < 0.05, ns: not significant.

To investigate the impact of circRNA‐1572 and bta‐miR‐2478‐L‐2 on the developmental potential of porcine IVF embryos, the blastocyst rate was calculated on the seventh day post‐fertilization. Our findings revealed that, compared to the 29.3% blastocyst rate observed in the NC group, both circRNA‐1572 KD and bta‐miR‐2478‐L‐2 overexpression significantly decreased the blastocyst formation rate to 15.7% and 14.8%, respectively (*p* < 0.05). Importantly, supplementary mRNA of *CCNB2* successfully rescued this phenotype, resulting in a blastocyst rate of 26.7%, 28.1%, 26.6% in the Si‐circRNA‐1572 + *CCNB2* mRNA, bta‐miR‐2478‐L‐2+ *CCNB2* mRNA and Si‐circRNA‐1572 + bta‐miR‐2478‐L‐2 inhibitor group, respectively (**Figure** **5**a).

Differential staining of blastocysts can be used to assess blastocyst quality, where higher total cell numbers or higher inner cell mass (marked with SOX2)/trophectoderm (marked with CDX2) (ICM/TE) ratios indicate better‐quality blastocysts. The results showed no significant differences in the total cell number among the groups, all exhibiting initial differentiation of the ICM and TE. However, the ICM/TE ratio was higher in the NC group (*p* < 0.05), whereas it was significantly lower in the circRNA‐1572 KD and bta‐miR‐2478‐L‐2 overexpression groups. Importantly, this effect was alleviated after the supplementary mRNA of CCNB2 as shown in the Si‐circRNA‐1572 + CCNB2 mRNA, bta‐miR‐2478‐L‐2+ CCNB2 mRNA and Si‐circRNA‐1572 + bta‐miR‐2478‐L‐2 inhibitor group (Figure 5b,c). Subsequently, apoptotic DNA fragmentation was assessed via the TUNEL assay to evaluate the quality of blastocysts, and results showed that, compared to the 3.4% apoptosis index observed in the NC group, both circRNA‐1572 KD and bta‐miR‐2478‐L‐2 overexpression significantly increased the apoptosis index to 13.3% and 13.2%, respectively (*p* < 0.05). Importantly, supplementary mRNA of CCNB2 successfully rescued this phenotype, resulting in an apoptosis index of 4.3%, 4.0%, 4.2% in the Si‐circRNA‐1572 + CCNB2 mRNA, bta‐miR‐2478‐L‐2+ CCNB2 mRNA and Si‐circRNA‐1572 + bta‐miR‐2478‐L‐2 inhibitor group, respectively (Figure 5d,e). Therefore, circRNA‐1572 plays a pivotal role in the quality and potential of porcine IVF embryos by suppressing bta‐miR‐2478‐L‐2.

### 2.7 CircRNA‐1572 Regulates F‐Actin Distribution and Chromosome Segregation During the Initial Cleavages in Porcine IVF Embryos by Targeting CCNB2 via miR‐2478‐L‐2

**Figure 6:**
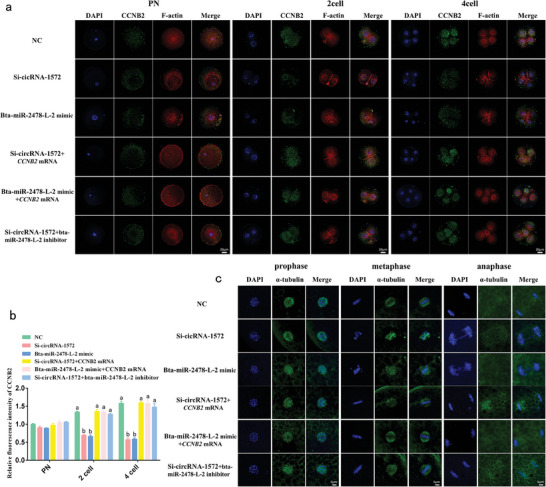
Sperm‐derived circRNA‐1572 regulates F‐actin distribution and chromosome segregation during early embryogenesis in porcine IVF embryos by targeting *CCNB2* via miR‐2478‐L‐2. a) Distribution of CCNB2 and F‐actin in the pronuclear (PN,1 pronuclear), 2‐, and 4‐cell embryos after injection of Si‐circRNA‐1572, bta‐miR‐2478‐L‐2 mimic, co‐injection of Si‐circRNA‐1572 + CCNB2 mRNA, co‐injection of bta‐miR‐2478‐L‐2 mimic + CCNB2 mRNA, co‐injection Si‐circRNA‐1572 + bta‐miR‐2478‐L‐2 inhibitor, and NC (n≥9 for each group, scale bar = 20 µm). b) Fluorescence intensity analysis of CCNB2. c)Following the injection of Si‐circRNA‐1572, bta‐miR‐2478‐L‐2 mimic, co‐injection of si‐circRNA‐1572 + CCNB2 mRNA, co‐injection of bta‐miR‐2478‐L‐2 mimic + CCNB2 mRNA, co‐injection of Si‐circRNA‐1572 + bta‐miR‐2478‐L‐2 inhibitor and NC, the dynamic changes of chromosomes and microtubules were assessed during the early stages of the first cleavage in porcine IVF embryos (a: prophase, b: metaphase, c: anaphase; *n* ≥ 9 for each group, scale bar = 5 µm). The data are presented as mean ± SEM from at least three independent experiments. different lowercase letters (a, b) indicate significant difference between groups, *p* < 0.05, ns: not significant.

Based on the crucial role of CCNB2 in mitosis and meiosis across various cell types, we hypothesized that the circRNA‐1572–bta‐miR‐2478‐L‐2–*CCNB2* axis regulates early cleavages in porcine IVF embryos, thereby affecting early embryonic development. Accordingly, we evaluated the expression, distribution, and morphology of CCNB2, F‐actin, and microtubules in early porcine IVF embryos. Immunofluorescence staining revealed that, in the NC group, CCNB2 was evenly distributed in the cytoplasm, with increasing fluorescence intensity from the PN to the 4‐cell stage (**Figure** **6**a,b). F‐actin filaments are evenly and densely distributed in the cytoplasm outside the nucleus (9/10) (Figure 6a, first row). In the Si‐circRNA‐1572 (Figure 6a, second row, circRNA‐1572 KD) and bta‐miR‐2478‐L‐2 mimic (Figure 6a, third row, bta‐miR‐2478‐L‐2 overexpression) groups, the fluorescence intensity of CCNB2 was significantly reduced at the 2‐ and 4‐cell stages. F‐actin filaments exhibited uneven and rough filamentous structures scattered outside the nucleus (7/9). Importantly, co‐injection of Si‐circRNA‐1572 + CCNB2 mRNA (Figure 6a, fourth row), co‐injection of bta‐miR‐2478‐L‐2 mimic + CCNB2 mRNA (Figure 6a, fifth row) and co‐injection Si‐circRNA‐1572 + bta‐miR‐2478‐L‐2 inhibitor (Figure 6a, sixth row) alleviated this effect in the majority of embryos (6/9, 7/9, 6/8, respectively). Additionally, during the metaphase of the first mitosis in porcine IVF embryos, the embryos in the Si‐circRNA‐1572 and bta‐miR‐2478‐L‐2 mimic groups displayed a distorted microtubule morphology and asymmetric bipolar spindles with atypical “spindle” shapes, and the chromosomes were dispersed and scattered on an equatorial plate (6/9 and 7/9, Figure 6c, second, third row, respectively). Further, co‐injection of Si‐circRNA‐1572 + CCNB2 mRNA (Figure 6c, fourth row), co‐injection of bta‐miR‐2478‐L‐2 mimic + CCNB2 mRNA (Figure 6c, fifth row) and co‐injection Si‐circRNA‐1572 + bta‐miR‐2478‐L‐2 inhibitor (Figure 6c, sixth row) alleviated this effect in the majority of embryos (7/10, respectively). Therefore, circRNA‐1572 modulates F‐actin distribution and chromosome segregation during early embryonic cleavages by targeting *CCNB2* through bta‐miR‐2478‐L‐2, thereby influencing the cell cycles in IVF embryos.

### 2.8 Transcriptome Analysis Revealed the Involvement of CCNB2 in Maternal Transcripts Degradation and ZGA During Porcine Embryogenesis

**Figure 7:**
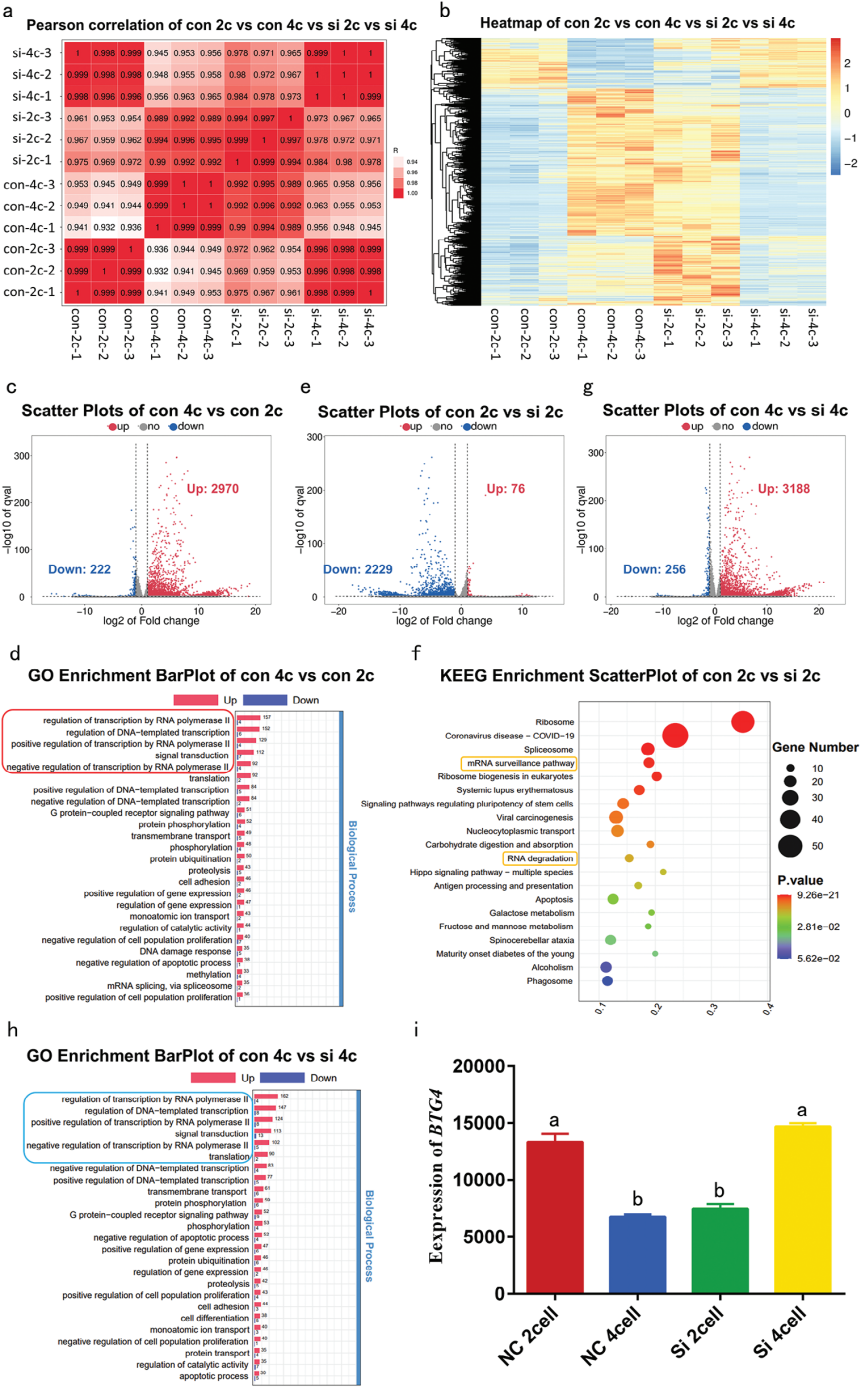
The Smart‐seq analysis revealed the involvement of CCNB2 in maternal mRNA degradation and zygotic genome activation during early porcine embryonic development. a) Pearson correlation analysis of the IVF 2‐ and 4‐cell embryos (NC group) and Si‐*CCNB2*‐injected 2‐ and 4‐cell embryos (KD group). b) Heatmap of differentially expressed genes in the 2‐ and 4‐cell embryos from the NC group and the KD group. c, e, j) Scatter plots showing the upregulated and downregulated gene expression in the 2‐ and 4‐cell embryos from the NC group, 2‐cell embryos from the NC group and the KD group, and 4‐cell embryos from the NC group and the KD group, with genes showing more than a twofold decrease or increase highlighted in blue and red, respectively. d) GO analyses of differentially expressed genes in the 2‐ and 4‐cell embryos from the NC group. f) KEGG analyses of differentially expressed genes in the 2‐cell embryos from the NC and KD groups. h) GO analyses of differentially expressed genes in the 4‐cell embryos from the NC and KD groups. i) Relative expression levels of circRNA‐1572 in NC group 2‐cell,4cell, Si‐CCNB2 group 2‐cell and 4‐cell (from RNA‐seq data, the data are presented as mean ± SEM, different lowercase letters a, b) indicate significant difference between groups, *p* < 0.05.

To further explore the mechanism underlying CCNB2 involvement in porcine ZGA, we collected normal (NC group) porcine IVF 2‐ and 4‐cell embryos as well as 2‐ and 4‐cell embryos injected with Si‐*CCNB2* (KD group) for SMART‐seq and analysis. Pearson correlation analysis revealed a high degree of correlation among the three samples of NC group 2‐ and 4‐cell embryos, as well as KD group 2‐ and 4‐cell embryos (**Figure** **7**a). Notably, compared to the NC 2‐cell stage, the gene expression pattern significantly increased in NC embryos at the 4‐cell stage, with 2970 upregulated and only 222 downregulated genes (Figure 7b,c). GO enrichment analysis revealed that the differential genes were mainly involved in the regulation of RNA polymerase II transcription, DNA template transcription regulation, and positive regulation of RNA polymerase II transcription (encircled by a red box, Figure 7d). KEGG enrichment analysis demonstrated that the differential genes were mainly related to ribosomes, ribosome biogenesis in eukaryotes, and RNA polymerase (Figure S2a, Supporting Information), indicating that extensive transcription commences at the 4‐cell stage, consistent with porcine ZGA.

Compared with the gene expression in the NC 2‐cell embryos, the KD 2‐cell embryos exhibited 76 (including *CCNB2*) downregulated genes and 2229 upregulated genes (**Figure** **7**b,e). Particularly, the number of upregulated genes in the KD group at the 2‐cell stage is significantly higher than the number of downregulated genes. GO enrichment analysis revealed that the upregulated genes were mainly involved in the nucleus, translation, and cytoplasmic ribosomes (Figure S2b, Supporting Information), while KEGG enrichment analysis showed that the differentially expressed genes were mainly related to mRNA surveillance pathway, and RNA degradation (encircled by the orange box, Figure 7f). Overall, these results suggest an impairment in maternal mRNA degradation and an accumulation of maternal mRNA in the KD group 2‐cell embryos, potentially leading to excessive translation of accumulated maternal mRNAs and consequent embryo division abnormalities.

**Figure 8:**
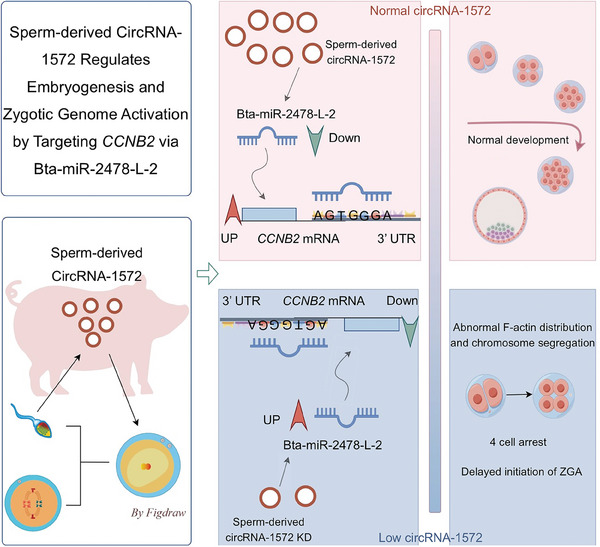
Model showing that sperm‐derived circRNA‐1572 regulates embryogenesis and zygotic genome activation by targeting *CCNB2* via bta‐miR‐2478‐L‐2.

Compared with the gene expression in the KD 4‐cell embryos, 3188 genes were upregulated (including *CCNB2* and the porcine ZGA marker genes *EIF1A* and *DPPA2*) and 256 genes were downregulated in the NC 4‐cell embryos (Figure 7b,g). Importantly, the gene expression profile of the KD 4‐cell embryos resembled that of the NC 2‐cell embryos, indicating a significantly higher overall transcription level in the NC group 4‐cell embryos than that of the KD group 4‐cell embryos. GO enrichment analysis revealed that the upregulated genes were mainly involved in the regulation of RNA polymerase II transcription, DNA template transcription regulation, and positive regulation of RNA polymerase II transcription (encircled by the blue box, Figure 7h), and KEGG enrichment analysis showed that the upregulated genes were mainly related to regulation of the actin cytoskeleton (encircled by the green box), ribosomes, and protein processing in the endoplasmic reticulum (Figure S2c, Supporting Information).

Noteworthily, *BTG4* (a mediator of mRNA clearance during the maternal‐to‐zygotic transition)^[^ ^33^, ^45^ ^]^ expression in the NC 2‐cell embryos was higher than that in both the NC 4‐cell embryos and Si‐*CCNB2* KD 2‐cell embryos, but comparable between the KD 4‐cell embryos and NC 2‐cell embryos (Figure 7i from RNA‐seq data). These results suggest that the NC group 2‐cell embryos underwent extensive degradation of maternal transcripts, resulting in a lower overall gene expression level, which was significantly increased at the 4‐cell stage, indicative of ZGA. In contrast, in the Si‐*CCNB2* KD group, the normal degradation of maternal transcripts was affected because of the significant reduction in CCNB2, leading to a higher overall gene expression level in 2‐cell embryos compared with the NC 2‐cell embryos, ultimately impeding ZGA in 4‐cell embryos.

## 3 Discussion

CircRNAs exhibit unique biological characteristics, such as widespread distribution, remarkable stability, and high specificity, enabling them to exert a crucial role in gene expression regulation. However, current knowledge regarding the function of circRNAs in mammalian embryonic development is still in its infancy, particularly regarding their interactions within complex regulatory networks alongside other sperm‐originating ncRNAs. Our study revealed that sperm‐derived circRNA‐1572 regulates early cleavages, ZGA, and embryonic developmental potential by acting as a “sponge” to adsorb bta‐miR‐2478‐L‐2 and target *CCNB2* in porcine IVF embryos (**Figure** **8**). This innovative exploration sheds light on the significant biological functions of circRNAs in early mammalian embryonic development and provides novel evidence on the role of sperm‐derived RNAs in maintaining and transmitting biological information across generations.

Spermatozoa after protamination contain both coding RNAs (mRNAs) and ncRNAs (such as miRNAs, lncRNAs, tRNAs, and circRNAs). The lack of transcriptional activity in the PN‐stage embryos may be responsible for the overall similarity in RNA expression profiles observed between MII oocytes and PN embryos in this study. Although previous studies have reported that sperm mRNA or ncRNA can directly enter oocytes to participate in embryonic development^[^ ^46^, ^47^, ^48^ ^]^ or indirectly influence early embryonic development through their involvement in epigenetic imprints,^[^ ^49^, ^50^ ^]^ these studies were restricted to only one or two types of samples, namely sperm, oocytes, or embryos. In contrast, our study stands out by simultaneously collecting three types of samples and rigorously selecting RNAs that were not expressed in oocytes but expressed in zygotes, and highly expressed in sperm. Initial research on sperm RNA mostly considered sperm RNA as a collective group (primarily miRNAs or tsRNAs), demonstrating their involvement in embryo development or the alteration of offspring phenotypes.^[^ ^51^, ^52^, ^53^ ^]^ However, subsequent studies have shown that individual RNAs (mainly mRNA, miRNA, or lncRNA) can also participate in embryonic development (such as regulating transcriptional activation, post‐transcriptional mRNA degradation and/or translation inhibition, or specific modifications) through unique regulatory mechanisms.^[^ ^54^, ^55^, ^56^, ^57^ ^]^ In accordance with this, we identified several porcine sperm‐specific miRNAs and circRNAs. qPCR results for six circRNAs in oocytes, sperm, and IVF embryos confirmed that circRNA‐1572, circRNA‐1892, and circRNA‐1881 are delivered into oocytes by sperm. In summary, our study, for the first time, revealed the significant role of sperm‐derived circRNAs in embryonic development compared with well‐known mRNAs, miRNAs, tsRNAs, and lncRNAs.

The abundance and stability of circRNAs, coupled with potential miRNA response elements within circRNAs, contribute to the “sponge” adsorption function of circRNAs for miRNAs, thereby playing significant roles in mammalian reproduction.^[^ ^58^, ^59^ ^]^ In this study, we identified, for the first time, sperm‐originated circRNA‐1572, which enters oocytes during fertilization. We hypothesize that the interaction between circRNA‐1572 and bta‐miR‐2478‐L‐2 in embryonic development is mediated through the “sponge” model, which was supported by subsequent experimental results. While research on the molecular regulatory mechanisms of circRNAs is ongoing, elucidating the mode of action in specific cell populations remains challenging due to difficulties in distinguishing circRNA from homologous linear RNA at multiple experimental levels. Nevertheless, the circular conformation endows circRNAs with significant regulatory potential for various biological activities. Unfortunately, due to the transcriptional pause in the fertilized egg before ZGA, overexpression plasmids of circRNA‐1572 cannot be efficiently expressed in the zygotes. Therefore, this study lacks relevant experiments on overexpressing circRNA‐1572 in parthenogenetic embryos or somatic cell nuclear transfer (SCNT) embryo models. However, as our additional data (Figure S4, Supporting Information) reveal, compared to IVF embryos, circRNA‐1572 is scarcely detected in parthenogenetic and SCNT embryos, and this further underscores the significance of sperm‐carried circRNA‐1572 in entering IVF embryos and its importance in the subsequent embryonic development. The widespread implementation of new cutting‐edge methods may facilitate studying or utilizing circRNAs in the future to explore the multifaceted mechanisms of circRNA regulation in embryonic development and disease occurrence.

The cytoskeleton is a complex network composed of microtubules, actin filaments, and intermediate filaments and it serves as a major component in embryonic morphogenesis.^[^ ^60^, ^61^, ^62^ ^]^ In early embryos, actin filaments and the microtubule system play a role in regulating spindle positioning, chromosome capture and separation, and cytoplasmic division.^[^ ^63^ ^]^ Increasing evidence supports a role for CCNB2 in mitosis, with studies showing its localization to kinetochores in HeLa cells and its interaction with MAD2 at the kinetochores to guide precise chromosome separation.^[^ ^64^ ^]^ Moreover, research has shown that the number of spindle pole misalignments in mouse embryonic fibroblast substantially increases compared with that in the equatorial plane during mid‐metaphase at high CCNB2 protein levels.^[^ ^65^ ^]^ Similarly, in our study, specific KD of sperm‐originated circRNA‐1572 significantly downregulated CCNB2 expression in porcine IVF embryos at the 2‐ and 4‐cell stages, leading to abnormal spindle formation and chromosomal mis‐segregation during the first mitotic division of zygotes and disorganized distribution of F‐actin in early embryos. Collectively, these findings support the role of CCNB2 in cell mitosis across various tissues. However, how CCNB2 participates in the mitotic signaling cascade during early mammalian embryo cleavage and whether it functions differently from its known roles in processes, such as kinetochore assembly, microtubule attachment, and separase activity, warrant further research and exploration.

It has been reported that the developmental arrest of mammalian in vitro embryos is often associated with ZGA,^[^ ^31^ ^]^ with the period of embryonic arrest aligning with the timing of ZGA.^[^ ^32^ ^]^ In many species, a major model for ZGA initiation is based on its regulation through changes in the ratio of nuclear to cytoplasmic components during the early cleavage cycles. The volume of embryos remains constant during the MZT, but the nuclear volume and nuclear content increase with each division cycle, a gradual increase in the nuclear‐to‐cytoplasmic ratio and subsequent early transcriptional activation.^[^ ^34^, ^45^ ^]^ Additionally, the normal cell cycle of embryos relies on the CDK1–Cyclin A/B‐mediated mitotic process.^[^ ^66^ ^]^ Interestingly, our results demonstrated that specific KD of sperm‐derived circRNA‐1572 and injection of bta‐miR‐2478‐L‐2 mimic downregulated *CCNB2* expression, resulting in arrest at the 2‐ and 4‐cell stage of porcine IVF embryos, leading to ZGA inhibition and compromised developmental potential of embryos. However, cell lineage staining of blastocysts revealed that targeted KD of circRNA‐1572 and injection of bta‐miR‐2478‐L‐2 mimic did not significantly alter the total cell number, but markedly reduced the ICM/TE ratio. This suggests that targeted KD of circRNA‐1572 appears to not affect the lineage differentiation and future placental formation of embryos, but rather impacts further fetal formation.

Moreover, in both humans and mice, BTG4 mediates maternal mRNA degradation by recruiting the CCR4–NOT complex to actively translated transcripts.^[^ ^67^ ^]^ Surprisingly, the results of our RNA‐seq revealed that *CCNB2* KD in the 2‐cell embryos led to delayed degradation of maternal transcripts and also delayed ZGA in the 4‐cell embryos, accompanied by downregulation of *BTG4*. Therefore, we inferred that the abnormal degradation of maternal mRNA caused by the specific KD of CCNB2 was closely associated with *BTG4* expression. This suggests that the pathway mediated by BTG4 for maternal mRNA clearance is highly conserved across mammals. Thus, the normal cell cycle (in which CCNB2 is involved), MRD, and ZGA during embryonic development are closely interconnected and mutually influential, and the complex and precise regulatory relationships among them, particularly the molecular functions of *CCNB2* and *BTG4* in the MZT, warrant further research. Overall, our study indicates that the regulatory pathway involving sperm‐derived circRNA‐1572 influences MRD, initiates ZGA, and ensures normal embryonic development.

In this study, we described previously undisclosed functions of sperm‐originated circRNAs during early IVF embryonic development. This study demonstrates that sperm‐derived circRNA‐1572 regulates early embryonic cleavages and ZGA in pigs through “sponge” adsorption of bta‐miR‐2478‐L‐2 targeting *CCNB2*. This regulation affects embryonic cleavages and ZGA, ultimately affecting early embryonic developmental potential. This study provides compelling evidence for the indispensable role of non‐DNA material in sperm in modulating early embryonic development, expanding our understanding of the initiation of individual life stages and epigenetic reprogramming regulation during early embryonic development. Further, our findings contribute to a deeper understanding of IVF technology and provide novel insights for improving technologies such as SCNT and parthenogenetic reproduction, which lack paternal genetic material. Additionally, this study demonstrates the pivotal contribution of paternal factors to embryonic development, thereby advancing our fundamental understanding of individual mammalian life forms.

## 4 Experimental Section

### Oocyte Collection and In Vitro Maturation (IVM)

Porcine follicular oocytes from Long White pigs were collected from ovarian follicles (3–8 mm in diameter) in postpubertal gilts suitable for breeding over 8 months old at a local slaughterhouse and transported to the laboratory at 38.5 °C in saline solution. The cumulus–oocyte complexes (COCs) were washed thrice with phosphate buffered saline (PBS) containing 5% fetal bovine serum (FBS, Gibco, BRL, Grand Island, NY, USA, A5669701). COCs with even cytoplasm and at least three layers of cumulus cells were selected for IVM. The culture medium for IVM comprised TCM‐199 bicarbonate buffer (Gibco, 11 150 059) with 1 mg mL^−1^ polyvinyl alcohol (PVA, Sigma‐Aldrich, USA, P8136), 1% (100×) insulin–transferrin–selenium (ITS, Gibco, 2 953 825), 0.91 mm sodium pyruvate (Sigma‐Aldrich, SLCJ1780), 3.05 mm glucose (Sigma‐Aldrich, G6152), 0.57 mm L‐cysteine (Sigma‐Aldrich, 168 149), 10 IU mL^−1^ equine chorionic gonadotropin (eCG, Ningbo Hormone Factory No.2, China, Cixi), 10 IU mL^−1^ human chorionic gonadotropin (HCG, Ningbo Hormone Factory No.2), 2.5 IU mL^−1^ follicle stimulating hormone (FSH, Ningbo Hormone Factory No.2), 40 ng mL^−1^ fibroblast growth factor 2 (FGF‐2, Sino Biological, Beijing, China, 10014‐HNAE), 20 ng mL^−1^ insulin‐like growth factor 1 (IGF‐1, Sino Biological,10598‐HNAE), 20 ng mL^−1^ leukemia inhibitory factor (LIF, Sino Biological, 14890‐HNAH), and 10 ng mL^−1^ epidermal growth factor (EGF, Gibco, PHG0311). Groups of 300 COCs were cultured in 3 mL medium in humidified air with 5% CO2 at 38.5 °C for 42–44 h.

### IVF and In Vitro Culture

In vitro fertilization was performed as previously described with some modifications.^[^ ^68^ ^]^ Groups of 30 COCs were transferred to 90 µL droplets of modified fertilization Tris‐buffered medium (mTBM) containing 10 mM Tris, 2 mg mL^−1^ bovine serum albumin (BSA, Sigma‐Aldrich, USA, A7030) and 2 mm caffeine. Fresh semen from Landrace boars aged 2 – 3 years purchased from the local breeding station and washed twice with TL‐HEPES (sperm washing solution) containing 2 mg mL^−1^ BSA. The sperm suspension was then added to PureSperm 40/80 (Nidacon, Sweden, Ps40‐100, Ps80‐100), balanced 3 h in advance for centrifugation, and the bottom sperm was washed with the sperm washing solution once. The supernatant was discarded, and the sperm was diluted with mTBM. Sperm activity was assessed on a cell counting plate before use, and 10 µL of sperm mTBM solution was added to the fertilization droplet. The final sperm concentration was 5 × 10^5^ sperm mL^−1^. After 6 h of COC/sperm co‐incubation, cumulus cells and sperm were vortexed for 4 min in PBS containing 5% Fetal Bovine Serum (FBS) and 0.1% (w/v) hyaluronidase (Sigma Aldrich, H3506) to remove cumulus cells and any excess surface sperm, and 50 fertilized oocytes with a second polar body were selected and transferred into 500 µL of porcine zygote medium 3 (PZM3), comprising 6.312 mg mL^−1^ NaCl (Sigma‐Aldrich, USA, S7653), 0.746 mg mL^−1^ KCl (Sigma‐Aldrich, P4504), 0.048 mg mL^−1^ KH2PO4 (Sigma‐Aldrich, P5655), 0.048 mg mL^−1^ MgSO4·7H2O (Sigma‐Aldrich, 63 138), 2.106 mg mL^−1^ NaHCO3 (Sigma‐Aldrich, S5761), 0.022 mg mL^−1^ sodium pyruvate (Sigma‐Aldrich, SLCJ1780), 0.617 mg mL^−1^ calcium lactate pentahydrate (Sigma‐Aldrich, C8356), 0.01 mL mL^−1^ (100×) glutamine (Sigma‐Aldrich, G6152), 0.546 mg mL^−1^ taurine (Sigma‐Aldrich, T8691), 0.02 mL mL^−1^ β‐mercaptoethanol (β‐ME, Sigma‐Aldrich, 63 689), 0.01 mL mL^−1^ minimum essential medium (MEM, Sigma‐Aldrich, 51411C), and 3 mg mL^−1^ BSA (Sigma‐Aldrich, USA, A7030). The oocytes were then cultured at 38.5 °C in 5% CO2 humidified air for subsequent microinjection, immunofluorescence staining, and RNA collection.

### Whole‐Transcriptome Sequencing and Analysis

The COCs were vortexed in PBS containing 5% FBS and 0.1% (w/v) hyaluronidase (hy, Sigma‐Aldrich, H3506) for 4 min to remove the cumulus cell contaminants. Total RNA was isolated and purified using Trizol reagent (ThermoFisher, 15 596 018) following the manufacturer's procedure from 10 000 porcine MII oocytes with no cumulus cells, mature purified fresh sperm of Large White boars (2‐to‐3‐year‐old), and 10000 PN embryos (18 h after the start of the IVF). The total RNA quantity and purity were analysis of Bioanalyzer 2100 and RNA 6000 Nano LabChip Kit (Agilent, CA, USA, 5067‐1511) with RIN number > 7.0. ≈5 µg of total RNA was used to deplete ribosomal RNA according to the manuscript of the Ribo‐Zero Gold rRNA Removal Kit (Illumina, cat. MRZG12324, SanDiego, USA). Then, the remaining RNA was fragmented using divalent cations (Mg^2^⁺ and Ca^2^⁺) under high temperature. The cleaved RNA fragments were then reverse‐transcribed into cDNA, which was then used to synthesize U‐labeled second‐stranded DNA with *Escherichia coli* DNA polymerase I, RNase H, and dUTP. An A‐base was added to the blunt ends of each strand, preparing them for ligation to the indexed adapters. Each adapter contains a T‐base overhang for ligating the adapter to the A‐tailed fragmented DNA. Single‐or dual‐index adapters were ligated to the fragments, and size selection was performed with AMPureXP beads. After the heat‐labile UDG enzyme treatment of the U‐labeled second‐stranded DNAs, the ligated products were amplified by PCR using the following conditions: initial denaturation at 95 °C for 3 min; 8 cycles of denaturation at 98 °C for 15 s, annealing at 60 °C for 15 s, extension at 72 °C for 30 s, and final extension at 72 °C for 5 min. The average insert size for the final cDNA library was 300 bp (±50 bp). Sequencing was performed using an Illumina NovaSeq 6000 (LC Bio Technology Co, China) following the manufacturer's protocol. EdgeR was used for differential (using thresholds |log2foldchange| ≥ 1 and q < 0.05), GO, and KEGG analyses. Raw data of all samples were uploaded to the Gene Expression Omnibus (GEO) of the National Center for Biotechnology Information (GSE288538 and GSE288539).

### Reverse Transcription and Quantitative Real‐Time PCR (qRT‐PCR)

Total RNA was isolated using TRIzol as previously described.^[^ ^69^ ^]^ A total of 1 µg of sperm RNA was incubated for 30 min at 37 °C with or without 3 U µg^−1^ RNase R (Epicentre Technologies, Wisconsin, USA, RNR071250) to verify the presence of circRNA, as previously described.^[^ ^70^ ^]^ For mRNA and circRNA, cDNA were synthesized using a PrimeScript II 1st Strand cDNA Synthesis Kit (Takara, Dalian, China, 6210A) from 1 µg of RNA and subsequently quantified using TB Green Premix Ex TaqII (Takara, RR820A). The miRNA cDNA was synthesized using a miRNA 1st StrandcDNA Synthesis Kit (by stem‐loop) (Vazyme, Nanjing, China, MR101‐01) and subsequently quantified using miRNA Universal SYBR qPCR Master Mix (Vazyme, MQ101). qRT‐PCR was performed following the manufacturer's instructions. Divergent primers were used to determine circRNA abundance. The relative expression of mRNA and circRNA were normalized to *GAPDH* and the relative expression of miRNA was normalized to *U6*. All primers used are listed in Tables S4–S6 (Supporting Information). The relative RNA expression levels were analyzed using the 2‐ΔΔCt method. All experiments were performed in triplicate.

### Microinjection

Microinjection was performed as previously described.^[^ ^71^ ^]^ The concentrations of double‐stranded Si‐circRNA‐1572, Si‐circRNA‐1892 (Ribobio, Guangzhou, China), Si‐CCNB2, bta‐miR‐2478‐L‐2 mimic, bta‐miR‐2478‐L‐2 inhibitor, in vitro transcribed mRNA of *CCNB2* (Sangon, Shanghai, China) and their respective negative control (NC) (Sangon, Shanghai, China) were adjusted to 20 µm for microinjection. All sequences are presented in Table S7 (Supporting Information). ≈2 µL of injection solution was loaded into a microinjection needle (Eppendorf, Hamburg, Germany). Embryos were transferred into PBS containing 0.2% PVA microdroplets and covered with mineral oil for injection 6 h after IVF initiation. Changes in the cytoplasm were closely monitored during the injection to ensure that the pulse pressure and injection volume of the experimental and NC groups were as consistent as possible. Injected embryos were then transferred into pre‐balanced PZM3 medium and cultured at 38.5 °C in 5% CO2 humidified air.

### Transfection of siRNA, Plasmids, and miRNA Mimics

CircRNA‐1572 specific siRNA and it's NC (designed and synthesized by RiboBio), overexpression plasmid for circRNA‐1572 (constructed by GeneSeed, Guangzhou, China), overexpression plasmid for *CCNB2* (constructed by Tsingke, Beijing, China), mmu‐mir‐3968‐l‐3‐1ss14AT, bta‐mir‐2478l‐2, ssc‐mir‐125a‐r‐1, and ssc‐mir‐125b mimics, and their respective NCs (designed and synthesized by Sangon) were transfected into fourth‐generation porcine fibroblasts (at a final concentration of 30 nm) using jetPRIME transfection reagent (Polyplus, 10 100 006, France) following the manufacturer's instructions. RNA and proteins were collected 36–48 h post‐transfection.

### Dual‐Luciferase Reporter Gene Assay

The predicted targets sequences of mmu‐miR‐3968‐L‐3‐1ss14AT, bta‐miR‐2478‐L‐2, ssc‐miR‐125a‐R‐1, and ssc‐miR‐125b in circRNA‐1572 and their corresponding mutations were designed and cloned into pSi‐Check2 along with luciferase reporter sequences as follows: downstream of the luciferase coding region, the 233‐bp circRNA‐1572 fragment containing the binding site 1 for mmu‐miR‐3968‐L‐3‐1ss14AT and bta‐miR‐2478‐L‐2 was inserted, which was called circRNA‐1572‐WT 1, and the luciferase plasmid containing the corresponding mutation sequence was called circRNA‐1572‐MUT 1. Additionally, downstream of the luciferase coding region, the 238‐bp circRNA‐1572 fragment containing the binding site 2 for ssc‐miR‐125a‐R‐1 and ssc‐miR‐125b was inserted, which was called circRNA‐1572‐WT 2, and the luciferase plasmid containing the corresponding mutation sequence was called circRNA‐1572‐MUT 2. Similarly, the predicted target sequences of bta‐miR‐2478‐L‐2 in the 3′ UTR of *CCNB2* (*CCNB2* WT) or its corresponding mutations (*CCNB2* MUT) were cloned into pSi‐Check2 along with luciferase reporter sequences.

The above luciferase reporter plasmids, mmu‐miR‐3968‐L‐3‐1ss14AT mimic, bta‐miR‐2478‐L‐2 mimic, ssc‐miR‐125a‐R‐1 mimic, and ssc‐miR‐125b mimic were co‐transfected into porcine fibroblasts (1 µg DNA and 30 nm miRNA mimics and NCs per well in a 12‐well plate). The luciferase reporter assay was performed using a Dual‐Luciferase Reporter Assay System kit (Promega, Madison, WI, USA, E1910) in accordance with the manufacturer's instructions to detect luciferase activity 48 h post‐transfection. The reporter activity was determined as the ratio of firefly luciferase activity to Renilla luciferase activity.

### Biotin‐Coupled Probe RNA Pulldown Assay

CircRNA‐1572 (GAAACCAACCTGGTATCTCT) and NC (GCTATGACGGAACAGGTATT) probes were designed and synthesized by RiboBio. A biotin‐coupled probe RNA pulldown assay was performed as previously described.^[^ ^72^, ^73^ ^]^ Briefly, following the manufacturer's instructions (Bersinbio, Guangzhou, China, Bes5103‐01), standard lysis buffer was used to lyse porcine fibroblasts overexpressing circRNA‐1572. The cell lysates were then incubated with streptavidin‐coated magnetic beads bound with biotin‐labeled circRNA‐1572 probe or NC probe at 37 °C for 3 h, followed by washing with the wash buffer. The mixture was incubated with proteinase K and lysis buffer at 55 °C for 1 h. Finally, the bound RNA was extracted and purified and reverse‐transcribed into cDNA, followed by the quantification of circRNA‐1572, mmu‐miR‐3968‐L‐31ss14A T, and bta‐miR‐2478‐L‐2 expression levels by q‐PCR.

### RNA‐Fluorescence In Situ Hybridization (FISH) Assay

The MII oocytes and PN embryos (18h) were incubated with 1% streptomycin protease (protease, Sigma‐Aldrich, P0652) for ≈1 min, followed by gentle pipetting to remove the zona pellucida. Subsequently, the embryos were washed 3 times with PBS. RNA FISH detection was performed according to previous studies^[^ ^73^, ^74^ ^]^ and the instructions provided in the GENESEED circRNA/miRNA in situ hybridization detection kit (GeneSeed, Guangzhou, China, H0105). Briefly, the zona‐free MII oocytes and PN embryos were fixed in 4% paraformaldehyde for 20 min at 25 °C and washed twice with PBS. Then, the samples were sequentially incubated at 25 °C with Solution A, B, C from the kit for 20, 15, and 15 min, respectively, and washed with PBS for 5 min each time, followed by a 15‐min incubation in 4% paraformaldehyde. The samples were then pre‐hybridized with 100 µL circRNA/miRNA Hybridization Buffer at 50 °C for 30 min and then transferred to droplets of hybridization buffer containing DIG‐labeled circRNA‐1572 and TYE563‐labeled bta‐miR‐2478‐L‐2 probes (QIAGEN, Germany) with a final probe concentration of 10 nm and hybridized overnight at 42 °C. The next day, the samples were washed with the kit's Washing Buffer at 42 °C for 2 min and 25 °C for 8 min. Subsequently, the samples were incubated with the kit's Blocking Buffer I for 1 h at 37 °C. The PBS‐washed samples were then incubated with anti‐Digoxin, FITC‐Conjugate under dark conditions at 25 °C for 2 h. After another PBS wash, the samples were stained with 4,6‐diamidino‐2‐phenylindole (DAPI) for 8 minutes to visualize the DNA. Finally, images were captured using a confocal microscope equipped with Airyscan 2 (Zeiss LSM 980).

### Immunofluorescence Staining of Embryos

The embryos were washed thrice with PBS containing 0.2% PVA (PBS/PVA) and fixed with 4% paraformaldehyde (Beyotime, Shanghai, China, P0098) in a culture dish overnight at 4 °C. After washing with PBS/PVA thrice for 5 min each, they were transferred into PBS/PVA containing 0.1% Triton X‐100 for permeabilization for 30 min at 25 °C. After washing, the embryos were incubated in Immunol Staining Blocking Buffer (Beyotime, P0102) for 3 h at room temperature. After washing, the embryos were incubated with anti‐CCNB2 (Abmart, Shanghai, China, TD9047S), phalloidin (Beyotime, C2203S), anti‐α‐tublin (abcam, England, ab52866), anti‐SOX2 (Cell Signaling Technology, USA, #3579), and anti‐CDX2 (BioGenex, USA, AM392‐5 m) overnight at 4 °C. After washing, the embryos were incubated with a secondary antibody (Alexa Fluor 488‐labeled goat anti‐rabbit IgG [1:500; Beyotime, A0423] and Alexa Fluor 555‐labeled donkey anti‐mouse IgG [1:500, Beyotime, A0460]) at 25 °C in the dark for 2 h. Subsequently, DNA was stained with DAPI (Beyotime, C1006) for 8 min. Finally, images were captured using a confocal microscope (Zeiss LSM 980 with Airyscan 2).

The Dead End colorimetric TUNEL system (Promega, G3250) was used to detect blastocyst apoptosis. The blastocysts were fixed overnight in 4% paraformaldehyde at 4 °C, permeabilized in 0.1% Triton X‐100 for 30 min at 25 °C, transferred into E buffer microdroplets at room temperature for 5 min, and incubated with FITC‐conjugated dUTP and terminal deoxynucleotidyl transferase at 37 °C for 1 h in the dark. To stop the reaction, 2× SSC (0.15 mol L^−1^ sodium chloride and 0.015 mol L^−1^ sodium citrate) was added, and the samples were allowed to stand for 15 min. The samples were then stained with DAPI at 25 °C in the dark for 8 min. The stained cells were observed under a confocal microscope (Zeiss LSM 980 with Airyscan 2).

EU incorporation assays were performed using BeyoClick EU‐488 (Beyotime, R0301S) to detect nascent RNA in 4‐cell embryos. Pig embryos at the 4‐cell stage (48 h post IVF initiation) were cultured in PZM3 containing 1 mm EU for 1 h prior to staining following the manufacturer's instructions. Images were captured using a confocal microscope (Zeiss LSM 980 with Airyscan 2).

### Western Blot

Western blot was performed as previously described,^[^ ^75^ ^]^ the total protein was extracted from porcine embryos (each group consisted of 200 embryos) or fibroblasts using RIPA lysis buffer containing a proteinase inhibitor (Beyotime, P10013). The protein were then boiled at 100 °C for 10 min. Proteins were separated using sodium dodecyl sulfate–polyacrylamide gel electrophoresis and transferred onto polyvinylidene difluoride (PVDF) membranes (Merck Millipore, USA). The PVDF membranes were then blocked using 5% nonfat milk and incubated with primary antibodies against CCNB2 (1:500, Abmart, TD9047S) at 4 °C overnight, followed by incubation with the corresponding secondary antibody(1:1000, Beyotime, A0208) at 37 °C for signaling detection. GAPDH served as the loading control. The gray values of the bands were calculated using the ImageJ software (National Institutes of Health, Bethesda, MD, USA).

### SMART‐Seq

Normal porcine (Si‐CCNB2 NC group) IVF 2‐ and 4‐cell embryos, and 2‐ and 4‐cell embryos injected with Si‐CCNB2 (KD group) were collected (three sets of samples for each type of embryo, with 10 embryos in each set). RNA was extracted from the samples using a single‐cell SMART‐seq lysis buffer. A modified oligo(dT) primer (SMART CDS Primer) was used for the first‐strand synthesis reaction. When SMARTScribeTM (Invitrogen, cat. 639 537, USA) reverse transcriptase reaches the 5ʹ end of the mRNA, it adds a few additional nucleotides to the 3ʹ end of the cDNA through its terminal transferase activity. The carefully designed SMARTer oligonucleotide pairs with a non‐template nucleotide and creates an extended template to enable SMARTScribe RT to continue replicating to the end of the oligonucleotide. The resulting full‐length, single‐stranded (ss) cDNA contains the complete 5ʹ end of the mRNA as well as sequences that are complementary to the SMARTer oligonucleotide. sscDNA was amplified using LD PCR and sufficient dscDNA was obtained for library construction.

cDNA was fragmented by incubating with dsDNA fragmentase (NEB, M0348S) at 37 °C for 30 min. Library construction began with the construction of the fragmented cDNA. Blunt‐end DNA fragments were generated using a combination of fill‐in reactions and exonuclease activity, and the desired fragments were selected using the provided sample purification beads. An A‐base was then added to the blunt ends of each strand, indexed Y‐adapters were ligated to the fragments, and the ligated products were amplified using PCR. Next, we performed paired‐end sequencing on an Illumina NovaseqTM 6000 (LC Sciences, USA) following the manufacturer's protocol. DESeq2 was used for differential (using thresholds |log2foldchange| ≥ 1 and q < 0.05), GO, and KEGG analyses. Raw data of all samples were uploaded to the Gene Expression Omnibus (GEO) of the National Center for Biotechnology Information (GSE288540).

### Statistical Analysis

Data were averaged from at least three independent experiments. Statistical analyses were performed using GraphPad Prism 9.0. The data are presented as the mean ± standard error of the mean (SEM). An unpaired Student's t‐test was used to compare two groups, whereas a one‐way analysis of variance followed by Tukey's post‐hoc test was used to compare multiple groups. Statistical significance was set at asterisks (“*”*p* < 0.05, “**” *p* < 0.01, “***” *p* < 0.001, “****” *p* < 0.0001) or various lowercase letters (a, b, and c, *p* < 0.05), and with ns indicating not significant. Immunofluorescence intensity was quantified using the ImageJ software (National Institutes of Health). The mean gray value (mean) was calculated using the following formula: integrated density (IntDen) /area.

## Conflict of Interest

The authors declare no conflict of interest.

## Author Contributions

Y.W. and Y.W. contributed equally to this study. Y.W., Y.W., Y.W., and Y. Z. designed the experiments; Y.W., Y.W., Y.L., Y.D., H.L., X.W., and Z.W. performed the experiments; Y.W. analyzed the results and wrote the paper; Y.W., Y.W., and J.S. revised and proofread the paper.

## Supporting information



Supporting Information

Supporting Information

Supplemental Movie 1

## Data Availability

The data that support the findings of this study are available from the corresponding author upon reasonable request.
