# Supplementary material for: Sperm‐Derived CircRNA‐1572 Regulates Embryogenesis and Zygotic Genome Activation by Targeting CCNB2 via Bta‐miR‐2478‐L‐2
Source: Adv Sci (Weinh). 2025 Mar 17;12(18):2414325. doi: 10.1002/advs.202414325 (PMC12079451; doi:10.1002/advs.202414325)
Supplement: Supplementary file 1 — Supporting Information [file ADVS-12-2414325-s002.docx]

**Supporting figure 1**

**
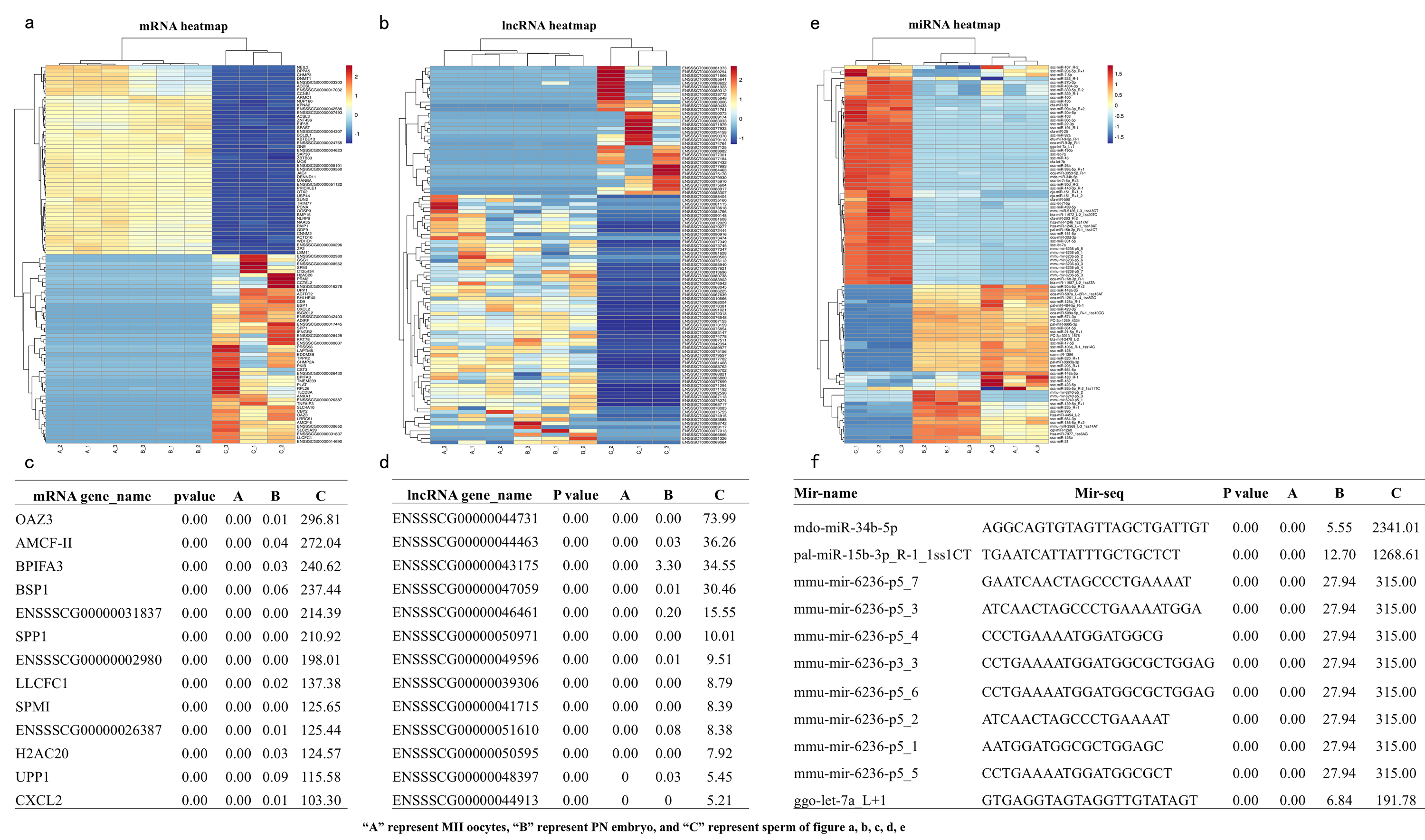
**

**
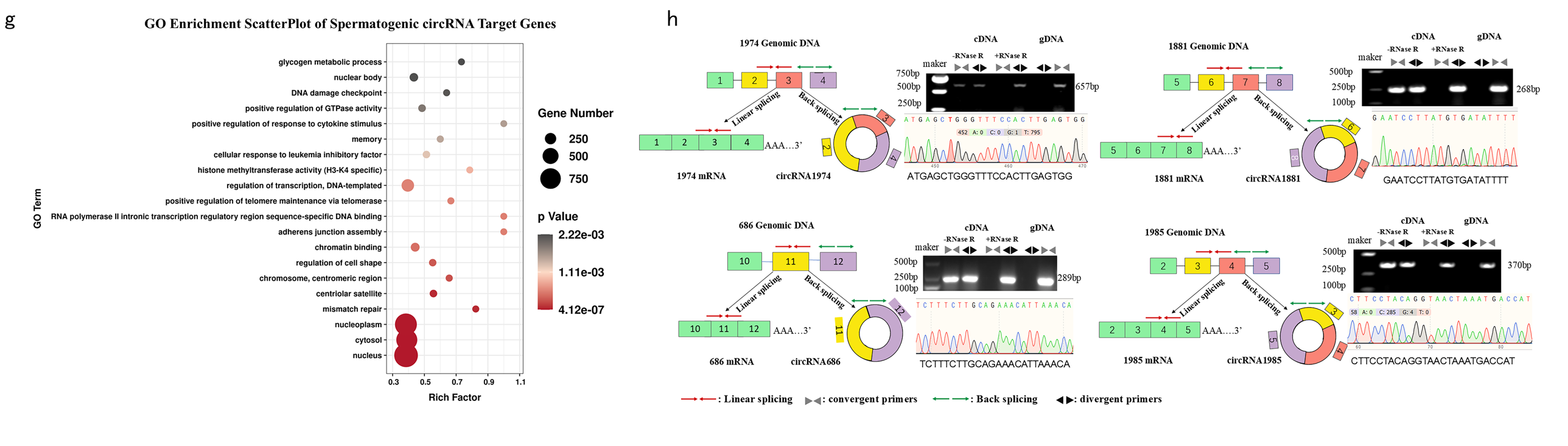
Supporting figure 1:** Identification of sperm-derived circRNAs. (a,c)Heatmap and expression of Mrna in porcine MII oocytes, PN embryos, and sperm. (b,d) Heatmap and expression of lncRNA in porcine MII oocytes, PN embryos, and sperm. (e,f ) Heatmap and expression of miRNA in porcine MII oocytes, PN embryos, and sperm. (g) GO analyses of Sperm-derived circRNA target genes (A, MII oocytes; B, PN embryos; C, sperm). (h) Genomic regions of the circRNA genes derived from various exons of circRNA-1881, circRNA-686, circRNA-1985, and circRNA-1974 (left). Convergent (gray) and divergent (black) primers used for amplification of linear or back-spliced products, RT-PCR of total RNA from RNase R-treated or untreated sperm (top right), and further validation through Sanger sequencing (bottom right)，all these demonstrated circular structure of the six circRNAs.

**Supporting figure 2
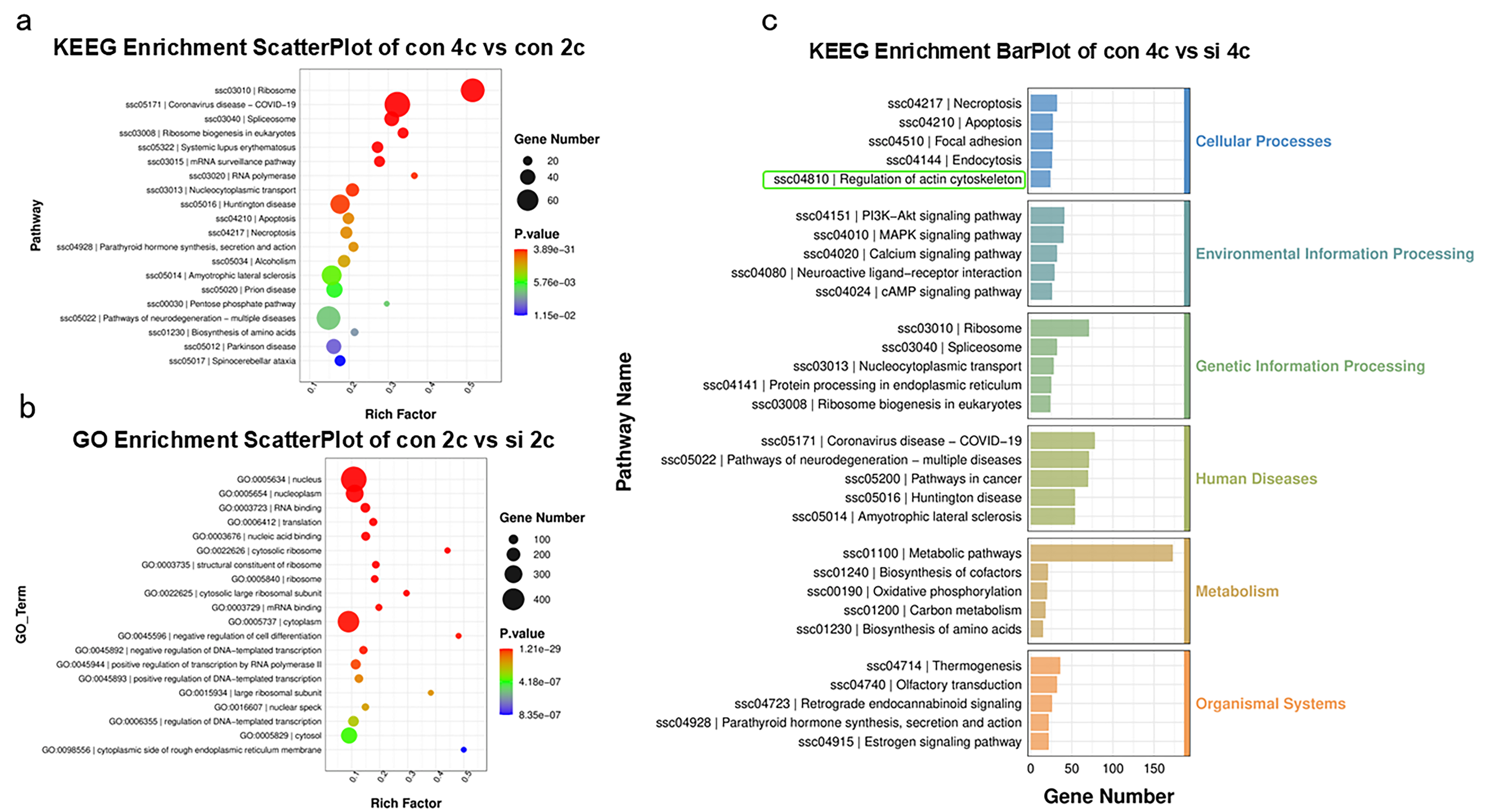
 Supporting figure 2**: The Smart-seq analysis. (a) KEEG analyses of differentially expressed genes in the 2- and 4-cell embryos from the NC group. (b) GO analyses of differentially expressed genes in the 2-cell embryos from the NC and KD groups. (c) KEEG analyses of differentially expressed genes in the 4-cell embryos from the NC and KD groups.

**Supporting figure 3**


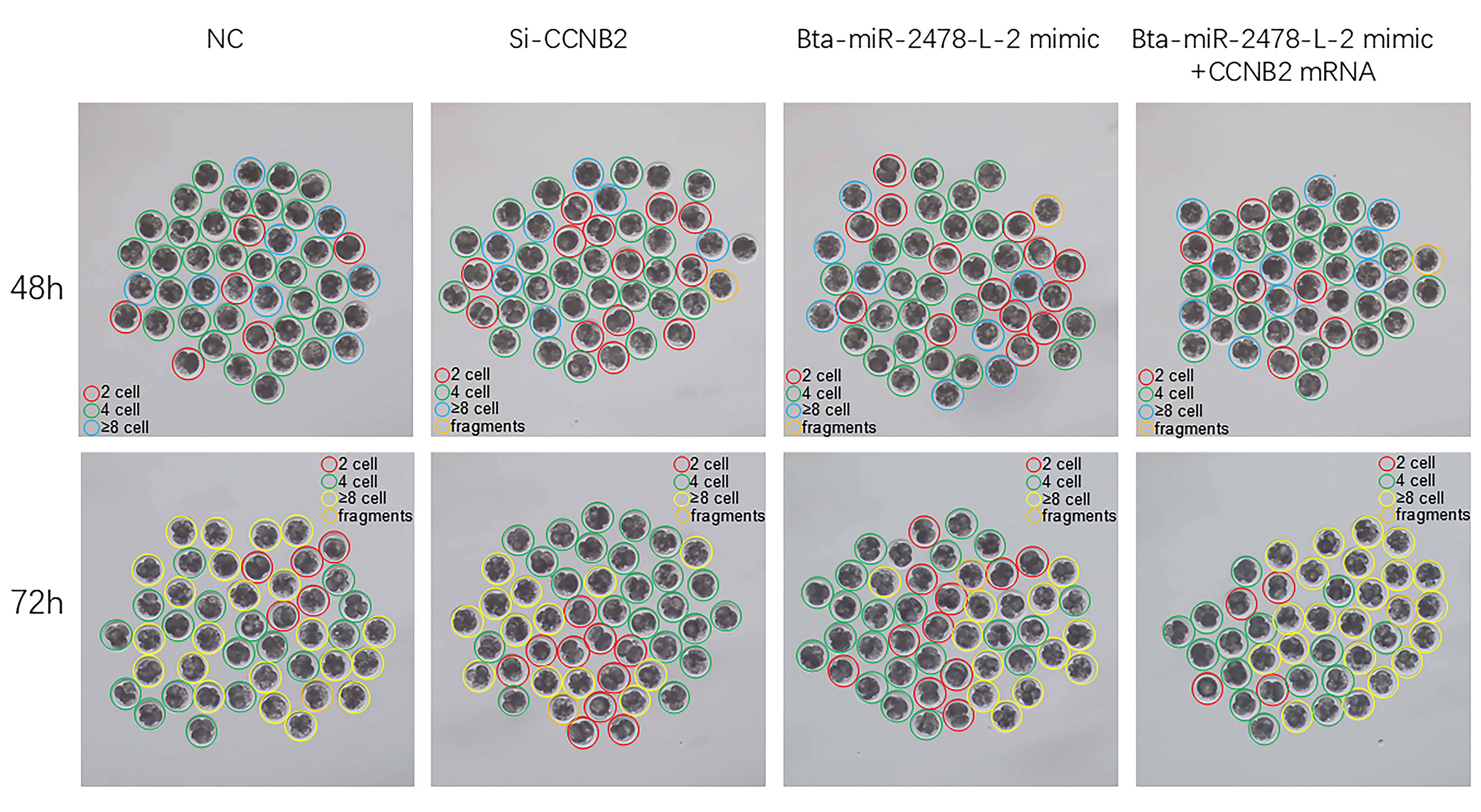


**Supporting figure 3**: Embryonic arrest at the 2- and 4-cell stages after injection of bta-miR-2478-L-2 mimic, Si-CCNB2 (KD group).

**Supporting table 1**: Sequence analysis and quality control of whole transcriptome sequencing

| **Sample** | **Raw Data** | **Valid Data** | **Valid Ratio(reads)** | **Q20%** | **Q30%** | **GC content%** |
| --- | --- | --- | --- | --- | --- | --- |
| A-1(MII oocytes) | 86100224 | 81146886 | 94.25 | 99.91 | 97.06 | 43.50 |
| A-2(MII oocytes) | 87133184 | 81931030 | 94.03 | 99.88 | 96.72 | 43.00 |
| A-3(MII oocytes) | 85993416 | 80886614 | 94.06 | 99.88 | 96.75 | 43.50 |
| B-1(PN embryo) | 86787850 | 79579650 | 91.69 | 99.92 | 97.37 | 42.50 |
| B-2(PN embryo) | 86477854 | 79975212 | 92.48 | 99.91 | 97.31 | 42.00 |
| B-3(PN embryo) | 84881926 | 79434200 | 93.58 | 99.91 | 97.22 | 43.00 |
| C-1(sperm ) | 84116752 | 78730376 | 93.60 | 99.92 | 97.10 | 45.50 |
| C-2(sperm ) | 85712242 | 80352870 | 93.75 | 99.92 | 97.13 | 45.50 |
| C-3(sperm ) | 85431206 | 79175646 | 92.68 | 99.92 | 97.13 | 45.50 |

**Supporting table 2**: Further selection of six circRNAs with biological information annotation.

| **Accession** | **Isoformname** | **Host Gene** | **Backreads** | **Circtype** | **circRNA size** | **Exon** | **gDNA size** |
| --- | --- | --- | --- | --- | --- | --- | --- |
| circRNA1881 | ENSSSCT00000061440 | PRKAR1A | 2091 | circRNA | 268bp | 6 7 8 | 1745bp |
| circRNA1974 | ENSSSCT00000030260 | ZSWIM6 | 1402 | circRNA | 657bp | 2 3 4 | 2555bp |
| circRNA1572 | ENSSSCT00000045747 | C18orf25 | 444 | circRNA | 785bp | 2 | 785bp |
| circRNA1892 | ENSSSCT00000019611 | MAP2K4 | 354 | circRNA | 398bp | 4 5 6 | 25765bp |
| circRNA1985 | ENSSSCT00000069681 | CREB1 | 319 | circRNA | 370bp | 3 4 5 | 15625bp |
| circRNA686 | ENSSSCT00000007372 | MAN1A2 | 261 | circRNA | 289bp | 11 12 | 4063bp |

**Supporting table 3:** The embryonic development after injection of si-circRNA1572 and si-circRNA 1892

| **Group** | **Cleavage at 28h after ivf** | | | **Cleavage at 48h after ivf** | | | | **Cleavage at 72h after ivf** | | | | | **Blastocyst at day 7 after ivf** | | |
| --- | --- | --- | --- | --- | --- | --- | --- | --- | --- | --- | --- | --- | --- | --- | --- |
|  | **1cell** | **≥2cell** | **total** | **1cell** | **2cell** | **≥4cell** | **total** | **2cell** | **4cell** | **≥8cell** | **total** | **Number of blastula** | | **rate** |  |
| NC | 46.95%^a^ | 53.05%^a^ | n=426 | 33.10% | 10.33%^a^ | 56.57%^a^ | n=426 | 12.63%^a^ | 38.25%^a^ | 49.12%^a^ | n=285 | 93 | | 32.63%^a^ |  |
| Si-cicRNA-1892 | 48.94%^a^ | 51.06%^a^ | n=378 | 34.39% | 9.79%^a^ | 55.82%^a^ | n=378 | 11.69%^a^ | 40.32%^a^ | 47.98%^a^ | n=248 | 74 | | 29.84%^a^ |  |
| Si-cicRNA-1572 | 58.40%^b^ | 41.6%^b^ | n=363 | 44.90% | 16.25%^b^ | 39.94%^b^ | n=363 | 18.00%^b^ | 50.00%^b^ | 32.00%^b^ | n=200 | 38 | | 18.5%^b^ |  |

**Supporting table 4**: Primers for the identification of circular structures in PCR

| **Genes** | **Forward (5′-3′)** | **Reverse (5-3′)** | **Annealing temperature (°C)** | **Product size (bp)** |
| --- | --- | --- | --- | --- |
| circRNA-1881 Convergent | TGATATTTTTGATGCCATGTTTCCGG | CATAAGGATTCTTCTGTAGCTGTCTCGG | 60 | 268 |
| circRNA-1881 Divergent | GAGAGACTGTTATTCAGCAAGGTGA | CAGCAATAAAGGAAACCGGAAACA | 60 | 268 |
| circRNA-1985 Convergent | GTAACTAAATGACCATGGAATCTGGAGCAGA | CTGTAGGAAGGCCTCCTTGAAAGAATTTC | 60 | 370 |
| circRNA-1985 Divergent | CCCAGCCATCAGTTATTCAGTCTC | CCGCCTGAATAACTCCATGGAC | 60 | 370 |
| circRNA-1974 Convergent | GTTTCCACTTGAGTGGCACAGTA | CCAGCTCATCCCAGAGCTGC | 60 | 657 |
| circRNA-1974 Divergent | GTGCTCCTGATCCAACAGCAGG | AGCCAGATCAGGTCAAATTGC | 60 | 657 |
| circRNA-1572 Convergent | GTTGGTTTCCTGTTGCATCATT | ATCTCTGCAGTGATTGTCTTGCT | 60 | 780 |
| circRNA-1572 Divergent | TGACCTGACTTGTGACTCAAGCA | CTATCACTGGTACTACTGTCCTGCA | 60 | 785 |
| circRNA-1892 Convergent | AAACGCAAAGCACTGAAGTTGAA | AGTGCGCCGTAAAACTGAACAA | 60 | 386 |
| circRNA-1892 Divergent | TTGGAGAAATTGGACGAGGAGC | GGTCTTTCAAGTCCTCTGCAGTG | 60 | 398 |
| circRNA-686 Convergent | AGCTAGGTCCTGAATCATTCAAGT | CTGCAAGAAAGAAGCTCTGCTGT | 60 | 273 |
| circRNA-686 Divergent | AAGTATTGCCGAGTCAGCAGT | TTCGATAGCCAGTGCTGCT | 60 | 386 |
| 1881(*PRKAR1A*) gDNA | GTGGCAGCTGGACAGAGTTCA | ACAGTTAAGACTCAAAACACAAGTGGAC | 60 | 268 |
| 1985(*CREB1*) gDNA | GCACTTATACCACCAACAGGAAAGC | CAGGTAGCTCCTTCACAGAATGACA | 60 | 370 |
| 1974(*ZSWIM6*) gDNA | ACAGACACAAGACACATACCACCAC | CCTCTAGCATGTTCTCTAATGAAGGGAA | 60 | 657 |
| 1572(*C18ORF25*) gDNA | GGATATGAACACTTTAAGGCTCT | TGTCAACTATCCCTACTGTGC | 60 | 786 |
| 1892(*MAP2K4*) gDNA | CCTCATGGATACTAGTCAGGCTT | GCATAAAACCTTAACTCCTGCTT | 60 | 398 |
| 686 (*MAN1A2*) gDNA | CATTTGCATGGAGTTTAACCGTA | ATGATTCAGATACACCTCCCGTA | 60 | 363 |

**Supporting table 5**: Primers for quantitative RT-PCR

| **Genes** | **Forward (5′-3′)** | **Reverse (5-3′)** | **Annealing temperature (°C)** | **Product size (bp)** |
| --- | --- | --- | --- | --- |
| circRNA-1881 | GTACACCTAGAGCAGCCACT | AGAAGTTATCCCCTTCATCACCT | 60 | 169 |
| circRNA-1985 | TTTCAACTATTGCAGAAAGCGAAG | TGTTACAGCTGCATCTCCAC | 60 | 162 |
| circRNA-1974 | TGACTGACAAATATAGGCAGCTC | GCGGTATAAAGACAATGCCACA | 60 | 206 |
| circRNA-1572 | GACTCAAGCACAAGCTCATCAGA | TTCAACTTTTCCCACTGCCTCC | 60 | 179 |
| circRNA-1892 | CTTCAGAGAGGTAAACGCAAAGCA | CTTCAGAGAGGTAAACGCAAAGCA | 60 | 241 |
| circRNA-686 | GCTATCGAAAAGTATTGCCGAGTCA | CACAGCCTCTACTGCACCA | 60 | 169 |
| *EIF1A* | GGTGTTCAAAGAAGATGGGCAAGAG | TTTCCCTCTGATGTGACATAACCTC | 60 | 115 |
| *DPPA2* | CCGTTCCTGCTTCTGTTGAGACC | GGCGAACCCAACCTTCTGTATCTG | 60 | 105 |
| *ZSCAN4* | GCCCAGAAAGTCTTCCCATGTGAG | GCCTCTCATCATTGTGTCTCCTCTG | 60 | 94 |
| *CCNB2* | ATCACAGACAACGCTTATACCAG | ACTTCATTCTCGGTGTATCCAGT | 60 | 314 |
| *GAPDH* | GCTTTTAACTCTGGCAAAGTGGAC | ACATACGTAGCACCAGCATCACC | 60 | 221 |

**Supporting table 6**: Primers for miRNA Stem loop reverse transcription and quantitative RT-PCR.

| **Genes** | **Stem loop reverse transcription** | **Forward (5′-3′)** | **Reverse (5-3′)** |
| --- | --- | --- | --- |
| bta-miR-2478-L-2 | GTCGTATCCAGTGCAGGGTCCGAGGTATTCGCACTGGATACGACTGGTGT | GCGCGGTATCCCACTTCTG | AGTGCAGGGTCCGAGGTATT |
| mmu-miR-3968-L-3-1ss14AT | GTCGTATCCAGTGCAGGGTCCGAGGTATTCGCACTGGATACGACTGGTGT | GCGCGAATCCCACTCCAG | AGTGCAGGGTCCGAGGTATT |
| U6 | GTCGTATCCAGTGCAGGGTCCGAGGTATTCGCACTGGATACGACGCTAAT | CGCGCGACGATACAGAGAAG | AGTGCAGGGTCCGAGGTATT |

**

 Supporting table 7**: Chemical synthesis of RNA sequences Supporting figure 4

| **Genes** | **sequence** |
| --- | --- |
| Si-circRNA-1892-1 | CACUCUUCAGAGAGGUAAA |
| Si-circRNA-1892-2 | GAGAGGUAAACGCAAAGCA |
| Si-circRNA-1572-1 | GCAGAGAUACCAGGUUGGU |
| Si-circRNA-1572-2 | GAUACCAGGUUGGUUUCCU |
| Si-CCNB2-1 | GCAGGUCCACUCCAAGUUU |
| Si-CCNB2-2 | GCUUAUACCAGUUCCCAAA |
| Bta-miR-2478-L-2 mimic | AUCCCACUUCUGACACCA |
| Bta-miR-2478-L-2 inhibitor: | UGGUGUCAGAAGUGGGAU |
| NC | CAG UAC UUU UGU GUA GUA CAA |
| mmu-miR-3968_L-3_1ss14AT mimic | AUCCCACUCCUGACACCA |
| ssc-miR-125a_R-1 mimic | UCCCUGAGACCCUUUAACCUGU |
| ssc-miR-125b mimic | UCCCUGAGACCCUAACUUGUGA |

***

ns

**Supporting figure 4**: Relative expression levels of circRNA-1572 in oocytes and various embryo, with total RNA extracted and detected using RT-qPCR. (Expression was normalized to that of *GAPDH*, ***P < 0.01, ns: not significant. IVF: in vitro fertilization, SCNT: somatic cell nuclear transfer, PA: parthenogenesis).

**Supporting figure 5**


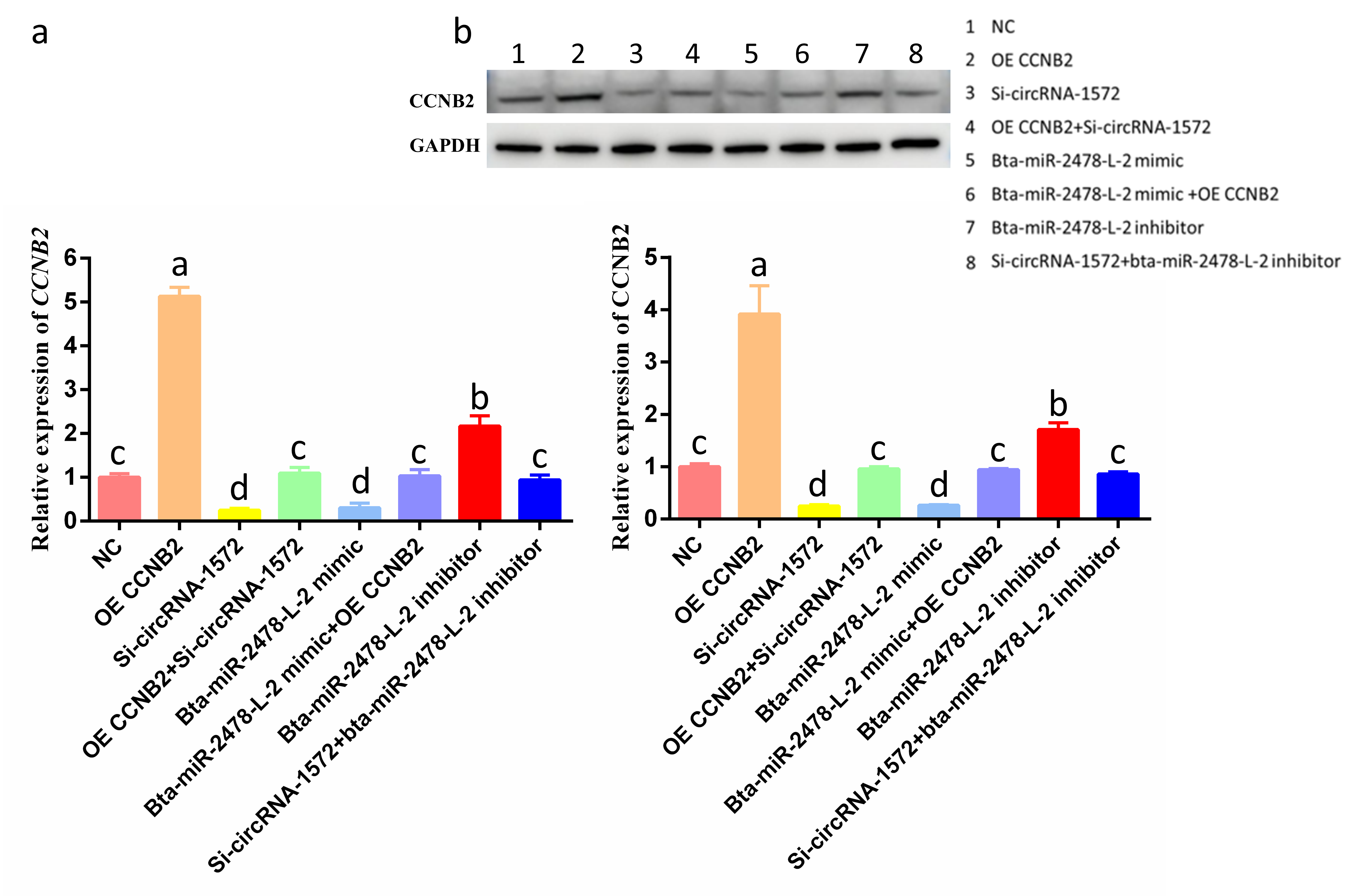


**Supporting figure 5**: Analysis of CCNB2 mRNA（A）and protein (B) expression levels in porcine fibroblasts after transfection of over expression (OE) CCNB2, Si-circRNA-1572, Si-circRNA-1572 + OE CCNB2, bta-miR-2478-L-2 mimic, bta-miR-2478-L-2 mimic + OE CCNB2, bta-miR-2478-L-2 inhibitor, or Si-circRNA-1572 + bta-miR-2478-L-2 inhibitor. (Expression was normalized to that of *GAPDH*, The data are presented as mean ± SEM from at least three independent experiments, different lowercase letters (a, b, c and d) indicate significant difference between groups, *P* < 0.05, ns: not significant).

1. circRNA-1572

GUUGGUUUCCUGUUGCAUCAUUGAUAUAGCUUCCUAGACUCUGCUCCAUGAUUUAAGUCAAAAGUCUGAAAUGAAGAUGGAGGAGGCAGUGGGAAAAGUUGAAGAACUCAUUGAGUCUGAAGUCCCACCCAAAACUUCUCAACAGGAGACAGCUAAGGAGGAAGAUGGAUCUGUAGAACUGGAAUCUCAAGUUCAGAAAGAUGGUGUGGUGGAUUCUGCAGUGCUUUCUUCAAUGCCCUGCUUGUUGAUGGAACUGAGAAGGGACUCUUCUGAGUCUCAGUUAGCAUCCACAGAGAGUGACAAGCCAACAACUGGCCGAGUUUAUGAGAGUGACUCCUCUAACCACUGCAUGCUUUCCCCUUCCUCUAGUGGUCACCUGGCCGAUUCAGAUACAUUGUCUUCUGCAGAGGAGAAUGAACCCUCCCAGGCAGAAACAGCGGUGGAAGGAGACCCUUCCGGAGUGUCUGGUGCCACAGUUGGGCGCAAGUCUCGCCGGUCCCGAUCUGAAAGUGAAACAUCCACCAUGGCUGCCAAGAAAAACCGGCAAUCCAGUGAUAAACAGAAUGGCCGAGUUGCCAAGGUUAAAGGUCAUCGGAGCCAAAAGCACAAGGAGAGAAUCAGGCUACUGAGGCAGAAACGAGAGGCUGCUGCACGGAAGAAAUAUAACCUGUUGCAGGACAGUAGUACCAGUGAUAGUGACCUGACUUGUGACUCAAGCACAAGCUCAUCAGAUGAUGAUGAAGAGGUUUCAGGGAGCAGCAAGACAAUCACUGCAGAGAUACCAG
